# Social network structure and depression by gender in rural Honduras: a cross-sectional study

**DOI:** 10.1136/bmjopen-2025-108285

**Published:** 2026-06-12

**Authors:** Selena T Lee, Marios Papamichalis, Karina Raygoza-Cortez, Nicholas A Christakis, Ana Lucia Rodriguez de la Rosa

**Affiliations:** 1 Harvard Medical School, Boston, Massachusetts, USA; 2 Human Nature Lab, Yale University, New Haven, Connecticut, USA; 3 Department of Sociology, Yale University, New Haven, Connecticut, USA; 4 Department of Statistics and Data Science, Yale University, New Haven, Connecticut, USA; 5 Department of Medicine, Yale University, New Haven, Connecticut, USA

**Keywords:** PUBLIC HEALTH, Postpartum Period, Depression, Postpartum, MENTAL HEALTH, Depression & mood disorders

## Abstract

**Abstract:**

**Objectives:**

To assess the relationship between the structural position of individuals within their village network and symptoms of depression and postpartum depression, among men and women.

**Design:**

Community-based, observational, cross-sectional study.

**Setting:**

176 villages in the Copan region of Honduras.

**Participants:**

Village residents, comprising 25 605 adults surveyed in a census-based study; using data collected between October 2015 and December 2019.

**Outcomes:**

Symptoms of depression and postpartum depression, among men and women.

**Results:**

Across all participants, 34.99% reported depression symptoms (40.50% for women and 27.62% for men). Among recent parents with a new child in the last 6 months, 28.89% reported postpartum depression symptoms (31.29% for women and 24.31% for men). Women with higher social intransitivity (ie, a greater proportion of friend-pairs among their friends that were not themselves connected) had higher odds of depression symptoms (OR=1.27, 95% CI 1.14 to 1.41), an association not found for men nor in postpartum parents. Because this coefficient is estimated on a 0–1 scale, it corresponds to approximately 2.4% higher odds of depression per 10 percentage-point increase in social intransitivity. In a signed-network decomposition that also included adversarial ties, only the proportion of incomplete/no-tie friend-pairs was associated with depression in women (OR=1.03, 95% CI 1.01 to 1.04), corresponding to approximately 3% higher odds of depression per 10 percentage-point increase.

**Conclusions:**

We report that structural social network position and connectedness beyond dyadic ties, including the friendships and adversarial ties of a person’s friends, are associated with depression. These findings highlight the importance of linking psychological health to broader social connections in the context of face-to-face relationships.

**STRENGTHS AND LIMITATIONS OF THIS STUDY**

- Analyses of a dataset with uncommon features (census and sociocentric data) for studying depression and village-wide social networks within an understudied, high-risk, resource-limited, rural population.
- Focus on higher-order network features such as triadic structures in face-to-face ties within the context of friendly and adversarial relationships, an uncommon approach to the study of depression and social networks.
- Gendered analysis to identify distinct factors positively and negatively associated with depression and a case study of postpartum parents (including men) in a non-clinical, community setting in a low- and middle-income country.
- Limitations include that data were collected in a specific region of a single country; that the outcome was collected as self-reported measures in the household context; and that the observational and cross-sectional nature of the study prevents us from making causal claims.

##  Introduction

Depression is among the most common psychiatric illnesses and a significant contributor to the global disease burden. Increases in its prevalence have positioned this condition as a leading cause of disability worldwide.^1^,^3^ Beyond its direct link to suicidal behaviour,^4^,^6^ depression is associated with increased mortality and poses a lifetime risk for a wide range of chronic illnesses,^7^,^11^ such as cardiovascular disease and cancer.^11^,^14^

The consequences of depression spill beyond affected individuals and are associated with the well-being and developmental outcomes of their families,^9 15^ communities and social networks.^16 17^ Likewise, research into social determinants of mental health has identified the inverse relationship: individuals’ social environments and networks significantly influence their risk of depression. Adverse social relationships (referred to as negative ties), poor community cohesion and resource-limited contexts in low- and middle-income countries (LMICs) have all been shown to increase risks of depression, especially for vulnerable groups, such as women, adolescents and older adults.^18^

In Latin America, estimates of depression are around 12%, more than twice the 5% global prevalence.^19^ The region’s history of community violence, high levels of poverty and inequality, food insecurity, relatively ineffective health systems and poor infrastructure have all been established as contributors to poorer mental health in these communities.^20 21^ Research and policy addressing the global depression crisis have emphasized the need for a multifactorial biopsychosocial framework attuned to changes across the lifespan, especially in such high-risk environments.^22^ In this framework, social networks—the complex web of community social ties, including both friendly and adversarial relationships—play a central role in understanding depressive symptoms.^23 24^ However, study designs involving the structural properties of community social networks (eg, using sociocentric designs in which all village ties are discerned) remain scarce in these populations.^2325^,^28^

### Social networks and depression

In high-risk settings such as LMIC, studies of community networks and depression are limited. However, research in other contexts has established at least two primary pathways linking social networks and depression: (1) the depression of one’s social contacts (alters) and (2) the individual’s structural position within the network, such as the degree of social isolation.^16 29 30^ Most research on social networks and depression uses an egocentric approach, in which surveys map the direct social niches of individuals (eg, by asking individuals sampled from a population who their friends are).

Among the few studies using sociocentric networks—mapping all social ties in a community—findings show that depression symptoms can spread through social ties beyond the second degree of separation^16^ and that certain structural arrangements within a network, such as transitivity (whether an individual’s friends are also friends with each other), affect the probability of suicidal thoughts.^31^ Some of these findings were gendered, such that women were more likely to influence a friend’s depression^16^ and were at increased risk for suicidal thoughts with increased intransitivity among their friends.^31^

When studying depression and social networks, most studies rely solely on friendship or familial relationships, namely positive ties. However, networks are naturally signed and include social relationships with people one does not get along with (negative ties), which remains understudied in the context of mental health. Most literature on signed graphs (networks with both positive and negative ties) have used egocentric designs to examine the relationship between mental health and social support, stress or familial relationships.^32^ Within this research, negative ties were associated with poorer psychological outcomes.^33^,^37^ Notably, these ties can be complex to study and interpret, particularly when considering negative and positive ties jointly.^38^ Novel research directions have uncovered increasingly complex structural properties of negative ties, such as the distribution of adversaries among one’s friends and the role they may play in shaping the topography of networks.^39^,^41^

### Gender, depression and postpartum depression

The relationship between social networks and psychological health, including depression and suicidal behaviour, has demonstrated gendered effects, such that women may be at increased risk of contagion and feelings of isolation, particularly due to different structural network arrangements.^16 31 42^ Overall, the literature is mixed on whether women or men have more supportive networks in relation to mental health. Some studies argue that while women report higher levels of social support, they are also at greater risk of negative interactions with their social ties, adversely affecting mental health.^43 44^ Furthermore, the types of social interactions differ by gender, with men less likely to seek emotional support than women, but more likely to report instrumental ties.^45^ Therefore, even when women’s networks might be reported as larger and more supportive, the nature of those networks (eg, positive vs negative ties) and the broader social structures beyond dyadic ties, can affect their mental health in ways that differ from men.^46 47^ For instance, in a classic study, American adolescent girls, but not boys, with more ties to non-mutual friends (ie, intransitive friendships) had increased suicidal ideation, often a symptom of depression.^31^

Gender also has a direct effect on depression symptoms. Cross-culturally and beyond social and economic variables, women, particularly older women, are at greater risk for depression and other mood disorders, except for substance misuse, of which men have a higher prevalence.^48^,^51^

The type of depression matters as well. Postpartum depression in women is strongly linked to hormonal changes during and after pregnancy, but may, secondarily, be exacerbated by changes in social connection and support.^52^ While there has been limited research on fathers experiencing depression in the postpartum period, a recent review on the topic shows that both genders should be considered when studying and intervening on postpartum families, even when the rates and predictors of paternal depression may differ.^53^

The present study seeks to contribute to the literature on social networks and depression by investigating sociocentric and signed graphs of individuals living in a resource-limited, rural, and LMIC setting. Controlling for established demographic correlates, we examine the structural properties (such as triadic characteristics) of signed village-wide social networks for 25 605 individuals living in 176 rural isolated villages in Copan, Honduras. Specifically, we focus on the role of negative ties and intransitive friendships in depression, studying men and women separately. Finally, we add insights into the differential correlates of depression and postpartum depression for both parents.

## Methods

### Study design

We conducted a cross-sectional analysis using data collected in a multiyear randomised controlled trial (RCT) in the region of Copan, in rural Western Honduras.^54^,^56^ The results of this parent study have been published elsewhere, and full details of the design and methods are reported in the study protocol.^55^ The purpose of this RCT was to assess social network targeting algorithms for population-level uptake following a 22-month educational intervention that counselled households on an array of practices related to maternal, child and neonatal health and other health outcomes.

### Recruitment and study sample

The parent study selected 176 villages in the Copan region. Recruitment for baseline census data prior to the RCT (‘Wave 0’ of data collection) was high, with 93% of the eligible population participating. At the beginning of the parent trial, 81.2% of those censused participated in survey data collection. Individuals aged 12 and older were eligible for enrolment in the baseline census survey, given the intervention’s topic and the local context of maternal health outcomes. However, only those aged 15 or older were part of the reproductive health survey (collected in ‘Wave 1’ and subsequent waves of data collection). Only individuals who were unable to provide consent or were younger than the eligible age range were excluded, as this was a census-based study. All adult participants provided verbal consent at the time of survey, and minors gave assent (with caregiver consent) before participating in any research activity. All participants were over 15 years old. For this study, only participants who completed all survey questions of interest (full description under ‘Participant selection’ in the online supplemental material) with no missing values were included. Across all waves, there were 25 605 unique eligible participants.

### Data source

**Figure 1:**
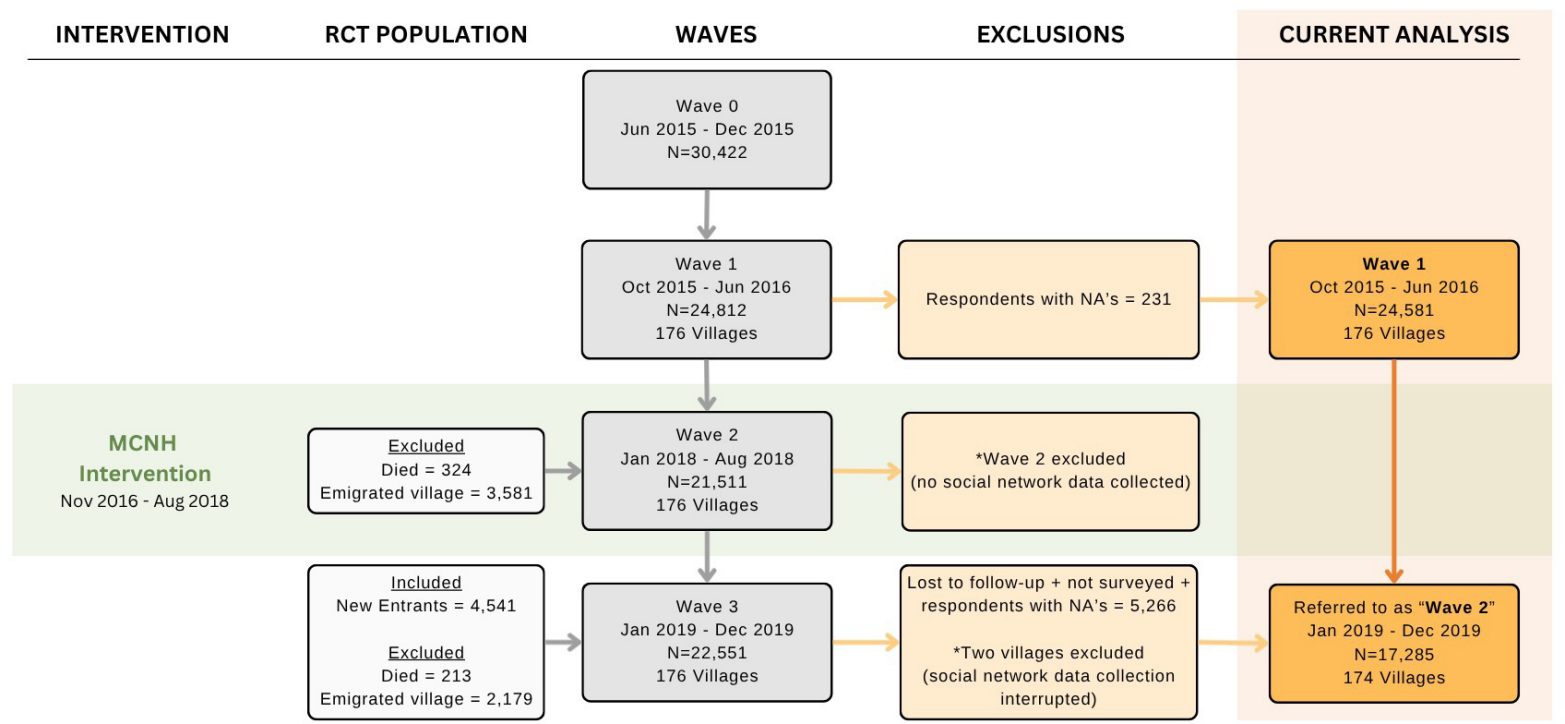
Data collection overview. Illustration of the data collection timing; MCNH intervention delivery for the original study, and sample sizes for this study, including sample size attrition over time with tracking of new entrants, emigration and deaths. NA, respondents with missing data; MCNH, maternal, child, and neonatal health; RCT, randomised controlled trial.

Data were collected between October 2015 and December 2019 in two waves of in-person surveys conducted in Spanish by a local team of highly trained research assistants. An overview of the data collection process is provided in figure 1. Surveys were household-based and lasted on average 45 min, including questions on social network ties, attitudes, behaviours related to maternal health and other physical and mental health outcomes. The timeline for data collection was the following: Wave 1 in 2015–2016 (N=24 581) and Wave 2 (labelled ‘Wave 3’ in the original study) in 2019 (N=17 285). In two villages, social network data collection was interrupted due to security concerns during Wave 2, so Wave 2 data from these villages were excluded from the analysis. Only survey questions present at all waves of data collection were used in this analysis. All measures remained consistent and in adherence with procedures established at the outset of the parent trial.

Given the multiyear nature of the data, we conducted a series of sensitivity checks in which each wave of data was analysed individually and in pooled models to study potential robustness across waves (online supplemental tables S2-S9).

### Outcomes: depression and postpartum depression

Participants were assessed for depressive symptoms using the PHQ-2 (Patient Health Questionnaire-2) survey.^57^ A validated scale, this short instrument is based on two questions: how often, over the prior 2 weeks, were participants bothered by: (1) little interest or pleasure in doing things, and (2) feeling down, depressed or hopeless. Responses were recorded on a 4-point scale, ranging from 0 = ‘not at all’ to 3 = ‘nearly every day’. A combined score across the questions of two or more indicated that major depressive disorder was likely, a recommended cut-off point in observational (non-clinical) high-risk settings to enhance sensitivity.^58^

To identify postpartum depression, participants of both genders who reported depressive symptoms and reported having a child younger than 6 months were coded as experiencing postpartum depressive symptoms. Adolescents who did not complete the full reproductive health history survey were excluded (N=2570). To be concise, we refer to depressive symptoms as ‘depression’ and postpartum depressive symptoms as ‘postpartum depression’.

### Social network construction

Sociocentric network studies map all relationships in a community, often via census surveys (in contrast to an egocentric approach). Sociocentric designs discern the direction and positive or negative nature of ties and model the community networks as reported directly by each member.

Our social network appraisal in each of the 176 study villages was conducted using Trellis software.^59^ At each wave, positive and negative ties were assessed. Friendships were identified through a standard set of so-called name-generator questions,^55^ asking participants to name those with whom they (1) ‘spent free time’, (2) considered to be ‘close friends’ and (3) discussed ‘personal and private matters’. Nominated individuals from the village who met any of these criteria were considered a positive tie. In turn, negative ties were assessed using a highly piloted, culturally responsive question in which participants identified village residents with whom they ‘did not get along’. Combining these data, we built a signed social network graph for each village and estimated all measures within village boundaries. Ties were symmetrised and simplified, meaning a nomination from any individual was considered bidirectional and counted once per positive (or negative) tie (eg, if person A names B as a friend or antagonist, we consider B a friend or antagonist of A).

### Independent variables: transitivity, intransitivity and negative triads

Our research aimed to examine the relationship between an individual’s depression and the social networks they were embedded in, focusing specifically on the triadic structures among their friends, particularly intransitive (vs transitive) friendships and also the particular types of triadic closure in signed networks (that afford not only positive, but also antagonistic ties). In a village network defined solely by friendships, social intransitivity reflects the degree of ‘non-closure’ among a participant’s friends—meaning two of an ego’s friends are not friends with each other (and are disconnected). Conversely, transitivity indicates the level of ‘closure’ among a participant’s friends. When networks include both positive and negative relationships, however, ‘non-closure’ can result from two different types of triads: (1) a participant’s friends may not be connected at all, indicating fragmentation within their friendship triad; or (2) a participant’s friends may be connected by a negative tie, indicating an imbalance within the triad.

**Figure 2:**
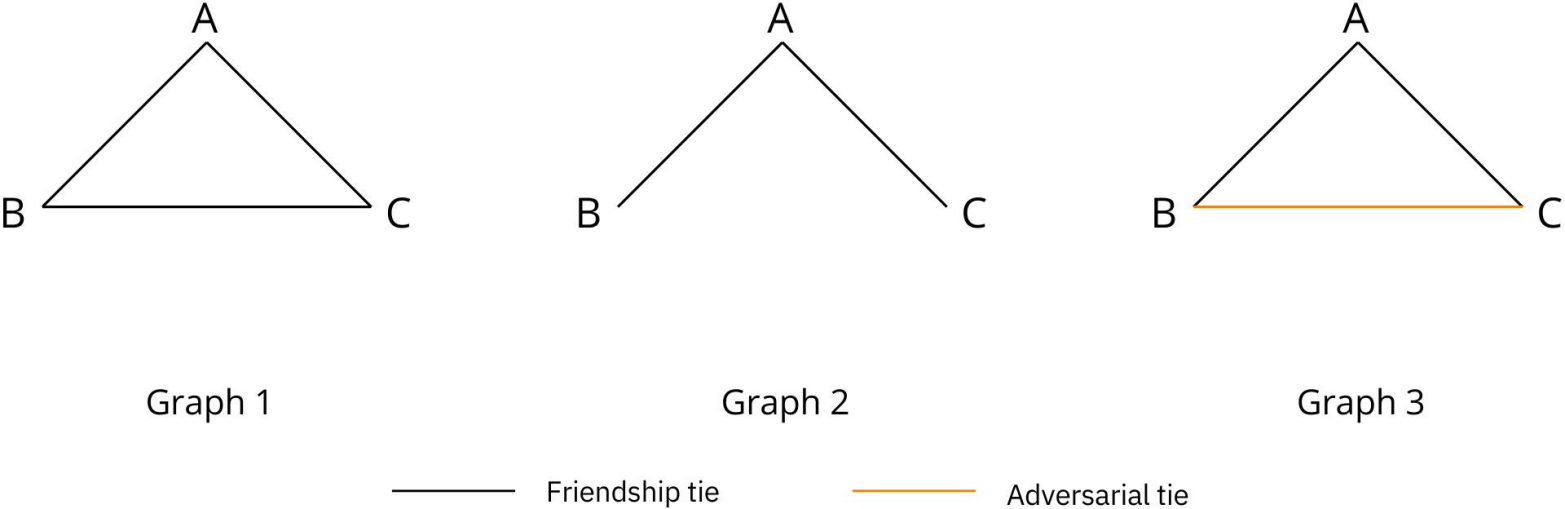
Triadic motifs. Each graph above represents a different scenario for three individuals in transitive or intransitive friendships in the context of signed social networks, where both friendly (positive) and adversarial (negative) ties are present. Here, friendships are represented as a black line connecting two individuals. Adversarial relationships are represented by an orange line. In Graph 1, individual A is friends with both B and C, who are also friends with one another. This case represents a balanced or transitive triad. In Graph 2, A is friends with both B and C, but B and C are not socially connected to one another, representing an incomplete or intransitive triad. In Graph 3, A is friends with both B and C, but B and C have an adversarial relationship, where at least one individual has identified the other as someone with whom they do not get along. This case represents a negative triad.

In our analysis, we first tested whether social intransitivity was associated with depression, and then whether this correlation was driven by a specific triadic motif (figure 2). To distinguish these structures, we decomposed individual-level intransitivity according to a participant’s relationship with their friends. For each individual (node) in the network with at least two friends, we classified each pair of their friends as (1) positive closure, forming a ‘balanced triad’ (ie, if they were friends), (2) negative closure, forming a ‘negative triad’ (ie, adversaries) or (3) non-closure, forming an ‘incomplete triad’ (having no tie in the positive or negative nominations) (figure 2).

We modelled the *proportions* of an ego’s friend-pairs in negative triads and incomplete triads, respectively, with transitive (or ‘balanced’) triads as the reference. This approach to characterising participants’ involvement in triadic motifs—represented as a proportional mixture of triad types, rather than individual counts—captures how the balance of closure, conflict and disconnection in a person’s friendship circle relates to depression. Alternatively, we also estimated and modelled the dominant triadic category in which each participant was involved and had similar results despite an alternate specification, indicating robustness of our findings (online supplemental table S16). These triadic measures capture broader social network traits, such as social isolation or discord among one’s friends, which may differentially affect a participant’s mental health. These triadic classifications are indicators of higher-order social realities.

### Explanatory variables and covariates

Demographic variables such as age, years of education, religion, marital status (partnered/unpartnered), ethnicity (indigenous identification) and food insecurity were included in all models as covariates. Likewise, household-level attributes included the number of cohabitants in the same building and the proportion of household members with depression. Village-level covariates included population size and geographical isolation (the number of access routes).

In addition, we included other social network covariates at both the individual (node) and village (network) levels. Node-level attributes were the number of friendship ties (positive degree), the number of adversarial ties (negative degree) and the percentage of depressed friends. We also averaged the friendship degree across each friend of a given participant (their neighbourhood’s degree). Global network attributes account for variation in network structures across villages. We estimated the density of ties in the village network, positive and negative. Density is the number of observed ties divided by the total number of possible ties among the nodes (participants in that village), and it measures connectedness.

Explanatory variables and covariates were included in every regression model. A detailed description of the data and coding is available in the online supplemental material, as well as the assessment of collinearity between variables, which was checked for all regression models. All model assumptions were checked, and we ran sensitivity analyses for each social network specification (online supplemental tables S10-S15).

### Statistical analysis

We used logistic regression with mixed effects to model associations with depression and postpartum depression cross-sectionally. We chose this approach to investigate the roles of intransitive friendships and triadic motifs in depression, controlling for social, demographic, household and village factors. All models were estimated separately for men and women, given expected differences in diagnosis, causes and manifestations of depression and postpartum depression.^47^,^5160^ All models accounted for individuals repeatedly surveyed across data collection waves (random intercepts for individuals with a fixed effect for the survey wave) and hierarchical structures in the data, clustering by household and village (random intercepts for each household and village). The reported coefficients for each model represent the average association between independent and dependent variables across the two waves. These models demonstrated robust goodness-of-fit (online supplemental material table S1). Using a likelihood ratio test to compare a null model with only random effects against the full model with fixed and random effects, the full model performed significantly better for both women ($\chi^{2}$= 2487.41, df=19, p<0.001) and men ($\chi^{2}$= 1597.83, df=19, p<0.001). Likewise, the postpartum model performed significantly better than a null model with only random effects for both women ($\chi^{2}$= 67.61, df=19, p<0.001) and men ($\chi^{2}$= 81.12, df=19, p<0.001).

**Table 1:** Demographic characteristics of study sample*

	All (N=25 605)†	Women (n=14 642)†	Men (n=10 963)†
Age, mean (SD)	32.99 (17.20)	32.89 (16.71)	33.12 (17.83)
Marital status			
Married/civil union	15 027 (58.69)	8878 (60.63)	6149 (56.09)
Not married/civil union	10 578 (41.31)	5764 (39.37)	4814 (43.91)
Postpartum, recent child in last 6 months	1689 (6.60)	1109 (7.57)	580 (5.29)
Depression symptoms, PHQ-2 ≥2	8958 (34.99)	5930 (40.50)	3028 (27.62)
Postpartum depression symptoms, PHQ≥2 and recent child in last 6 months	488 (1.91)	347 (2.37)	141 (1.29)
Education (years), mean (SD)	3.56 (2.93)	3.59 (2.96)	3.51 (2.88)
Religion			
Catholic	13 312 (51.99)	7897 (53.93)	5415 (49.39)
Protestant	8817 (34.43)	5562 (37.99)	3255 (29.69)
Indigenous	2969 (11.60)	1545 (10.55)	1424 (12.99)
Food insecurity			
Food insecure	11 392 (44.49)	6659 (45.48)	4733 (43.17)
Not food insecure	14 213 (55.51)	7983 (54.52)	6230 (56.83)
Number of household members, mean (SD)	3.09 (1.38)	3.09 (1.38)	3.09 (1.38)
Village size, mean (SD)	120.67 (78.15)	120.67 (78.15)	120.67 (78.15)
Access routes, mean (SD)	1.90 (0.73)	1.90 (0.73)	1.90 (0.73)

* Demographic characteristics were measured at baseline (initial survey) for each participant. Values in this table reflect characteristics recorded at this initial survey, whether the participant was first surveyed at Wave 1 or 2 of the study.

† Presented as n (%) unless otherwise noted.

PHQ-2, Patient Health Questionnaire-2.

To expand our understanding of the link between intransitive friendships and depression, we examined intransitive friendships within signed networks in the three scenarios: (1) balanced triads, (2) incomplete triads and (3) negative triads (figure 2). Across all waves of data collection, we analysed differences in depression for participants in relation to the proportion of their participation in balanced triads, as compared with incomplete or negative triads, while controlling for all covariates. We used a logistic regression with mixed effects for these comparisons, including a random intercept for individuals to account for repeated surveys across two time points. The model demonstrated goodness of fit (online supplemental material table S1) and performed significantly better than a null model with only random effects for both women ($\chi^{2}$= 2337.75, df=20, p<0.001) and men ($\chi^{2}$= 1569.75, df=20, p<0.001). All statistical analyses were conducted in R (V.4.4.2).

### Patient and public involvement

In the parent RCT, community leaders from each village served as liaisons and partners in all phases of the study. A focus group of leaders provided insight into depression and postpartum depression within their communities. The intervention and data collection teams actively engaged local communities at each stage of the RCT design to ensure feasibility and cultural responsiveness. The RCT was implemented as a community-centred ‘Lab in the Field’ study, and beyond the interviews, pilot surveys and other community engagement mechanisms, participants also received information on mental health hygiene. Furthermore, at the time this study is being written, ongoing efforts continue to be made to share research findings on social networks, health and mental health to many participant communities in town-hall-style meetings. Lastly, the Honduran Ministry of Health, the national policy health authority has been briefed and continues to support this and all ongoing research efforts.

## Results

We mapped the complete social networks of 176 rural villages in Honduras for 14 642 women and 10 963 men, using sociocentric network data on face-to-face friendships and adversaries to study depression among adults. We examined the relationship between depression and the structural position of individuals within their village network, namely social intransitivity, and the role of three types of triadic motifs present in participants’ positive and negative networks.

The distribution of all variables included in the models is listed in table 1. The study population was predominantly women (57.18%), Catholic (51.99%) and married or in a civil union (58.69%). Most participants did not identify as indigenous (88.40%). The average age was 32.99 years (SD 17.20). Educational attainment was low, with an average of 3.56 years of formal education (SD 2.93). Nearly half of the individuals experienced food insecurity (44.49%).

Overall, 34.99% of participants reported symptoms of depression, more than double the Latin American average. The proportion of depression symptoms was higher among women (40.50%) compared with men (27.62%). Notably, these high levels of depression symptoms remained mostly unchanged over time for both men and women (online supplemental table S2). Among parents who had a child in the prior 6 months (hereafter, referred to as ‘postpartum’, encompassing 1109 women and 580 men), one-third reported depression, with the same gendered pattern: 31.29% for women and 24.31% for men, and again with no statistically significant difference between waves (online supplemental table S2).

### Depression and associations with intransitivity and negative triads

**Figure 3:**
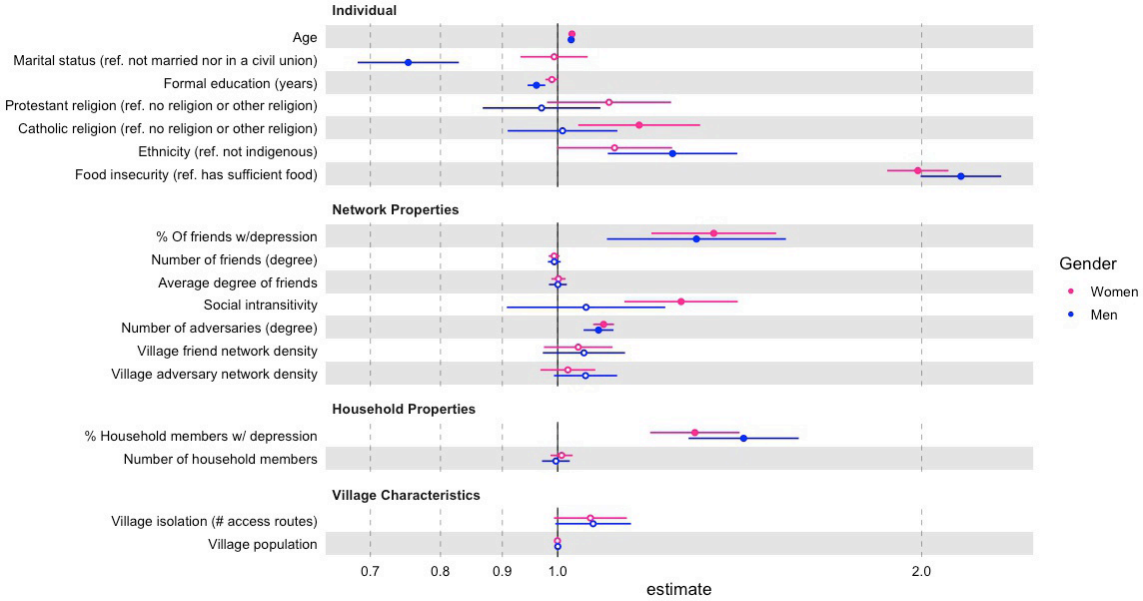
Mixed-effects logistic regression of depression. Each point represents an OR from the logistic regression models analysing depression for women (n=14 642, conditional R^2^=0.223) and men (n=10 963, conditional R^2^=0.208). Colour-filled points indicate significant values (p<0.05), and unfilled points (white) indicate non-significant values. The lines extending from each point demarcate the CI. The ORs and CIs are coloured by gender with pink representing values for women and blue for men.

When considering the measure of intransitivity while controlling for covariates (table 2), only non-postpartum women had higher odds of depression with greater social intransitivity (OR=1.27, 95% CI 1.14 to 1.41). Because social intransitivity was modelled on a 0–1 scale, its coefficient corresponds to a full one-unit increase on that scale; rescaled, the effect size is approximately a 2.4% increase in odds per 10 percentage-point increase in intransitivity. No association was found for men or postpartum parents (both genders). Figure 3 illustrates the ORs and 95% CIs for each variable included in the logistic regression models.

**Table 2:** Mixed-effects logistic regression table of individual and network characteristics on depression

Variable	Depression among participants					
	Men			Women		
	OR	(95% CI)	P value	OR	(95% CI)	P value
(Intercept)	0.09	(0.06 to 0.12)	<0.001	0.11	(0.08 to 0.14)	<0.001
Individual						
Age	1.03	(1.02 to 1.03)	<0.001	1.03	(1.03 to 1.03)	<0.001
Marital status (ref. not married nor in a civil union)	0.75	(0.68 to 0.83)	<0.001	0.99	(0.93 to 1.06)	0.838
Formal education (years)	0.96	(0.94 to 0.98)	<0.001	0.99	(0.98 to 1.00)	0.083
Religion (ref. no religion or other religion)						
Catholic	1.01	(0.91 to 1.12)	0.861	1.17	(1.04 to 1.31)	0.009
Protestant	0.97	(0.87 to 1.09)	0.592	1.10	(0.98 to 1.24)	0.104
Ethnicity (ref. not indigenous)	1.25	(1.10 to 1.41)	0.001	1.12	(1.00 to 1.24)	0.053
Food insecurity (ref. has sufficient food)	2.16	(2.00 to 2.33)	<0.001	1.99	(1.87 to 2.11)	<0.001
Household						
Number of household members	1.00	(0.97 to 1.02)	0.805	1.01	(0.99 to 1.03)	0.490
% household members w/depression	1.43	(1.28 to 1.58)	<0.001	1.30	(1.19 to 1.41)	<0.001
Village						
Village population	1.00	(1.00 to 1.00)	0.505	1.00	(1.00 to 1.00)	0.372
Village isolation (# access routes)	1.07	(1.00 to 1.15)	0.066	1.06	(0.99 to 1.14)	0.079
Social network						
Number of friends (degree)	0.99	(0.98 to 1.01)	0.311	0.99	(0.98 to 1.00)	0.213
% friends w/depression	1.30	(1.10 to 1.55)	0.002	1.35	(1.20 to 1.52)	<0.001
Number of adversaries (degree)	1.08	(1.05 to 1.11)	<0.001	1.09	(1.07 to 1.11)	<0.001
Average degree of friends	1.00	(0.98 to 1.02)	0.959	1.00	(0.99 to 1.02)	0.816
Social intransitivity*	1.06	(0.91 to 1.23)	0.482	1.27	(1.14 to 1.41)	<0.001
Village friend network density	1.05	(0.97 to 1.14)	0.210	1.04	(0.97 to 1.11)	0.239
Village adversary network density	1.06	(0.99 to 1.12)	0.084	1.02	(0.97 to 1.07)	0.466
Wave of data collection	1.03	(0.93 to 1.13)	0.615	1.06	(0.98 to 1.37)	0.167

* Social intransitivity is measured on a scale of 0–1, whereby a value of 1 indicates that 100% of a person’s friend-pairs are intransitive (not a single friend is connected to any other friend).

**Figure 4:**
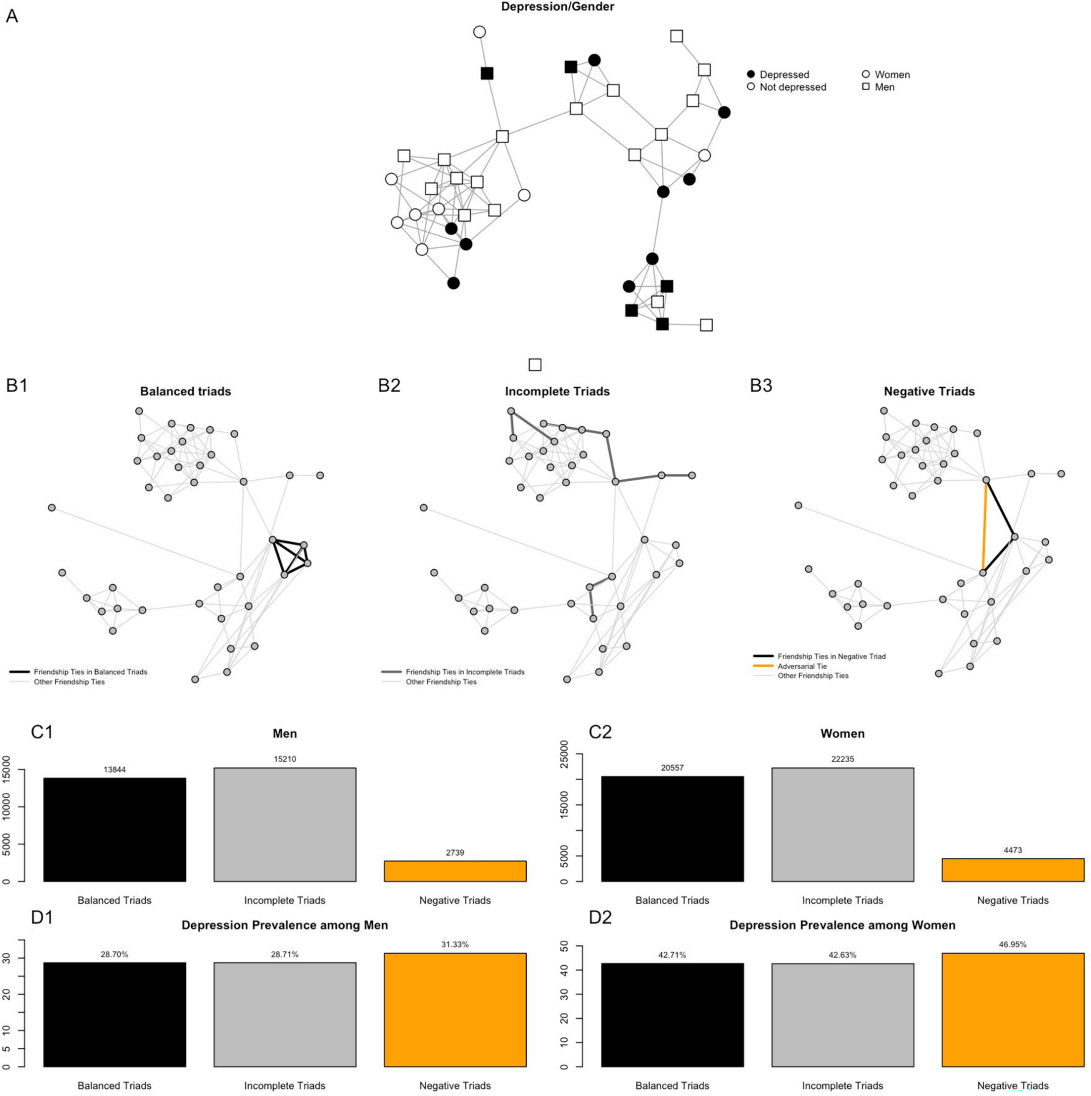
Triadic motifs and depression. (**A**) Social network of a representative village showing distribution of depression among residents. Depressed residents are indicated by black points (nodes), non-depressed residents by white points. Nodes are shaped according to gender (women are circles; men are squares). Lines (ties) between points represent friendships. (**B**) Each network is of the same village as in (**A**) and displays each of the three triad types illustrated in figure 2. (**B1**) This network highlights four balanced triads where all three members of a triad are friends with one another. (**B2**) This network illustrates several incomplete triads, where only two out of three possible friendships are present in a given triad. (**B3**) This network demonstrates a negative triad, where two out of three possible friendships are present in a triad, but the third relationship is negative. Among the two individuals in the negative relationship, at least one individual nominated the other as someone with whom the individual does not get along. (**C**) Prevalence of each triad type across all waves of data collection. Each bar corresponds to the number of instances (across all time points) of a participant involved in at least one of a given triad type for men (**C1**) and women (**C2**) across the study population. (**D**) Prevalence of depression among each triad type across all waves of data collection. Each bar corresponds to the proportion of men (**D1**) and women (**D2**) involved in at least one example of the given triad type expressing depression. Panels (**C**) and (**D**) are descriptive counts based on involvement in any triad type, whereas table 3 models the proportion of friend-pairs in each triad type.

Incomplete triads were the most common kind of triad, with 37 445 instances (across both time points) of participants involved in at least one incomplete triad, followed by balanced triads with 34 401 instances (figure 4). Negative triads were the least common, with 7212 occurrences. Incorporating signed social networks—where positive and negative relationships are present—added nuance to the foregoing results by decomposing different sources of intransitivity. Intransitivity may be caused by an adversarial relationship between a person’s friends, or it may be caused by a complete lack of association among friends. By including both possibilities within the same model, we were able to determine the main driver of association between intransitivity and depression among women. Initially, we observed differences in depression for participants in balanced triads as compared with those in negative triads (figure 4).

Though the unadjusted depression proportion was elevated among both men and women involved in negative triads, this association was not statistically significant after adjusting for covariates. In the signed-network decomposition (table 3), only involvement in a greater proportion of incomplete triads, in which a participant’s friends were socially disconnected from one another—neither expressing friendship nor animosity—was significantly associated with depression for women. Specifically, a 10 percentage-point increase in the proportion of incomplete friend-pairs was associated with 3% increased odds of depression among women (OR=1.03, 95% CI 1.01 to 1.04), whereas the negative component was not significant.

**Table 3:** Mixed-effects logistic regression table of triadic network characteristics on depression

Variable	Depression among participants					
	Men			Women		
	OR	(95% CI)	P value	OR	(95% CI)	P value
(Intercept)	0.09	(0.06 to 0.12)	<0.001	0.11	(0.08 to 0.14)	<0.001
Individual						
Age	1.03	(1.02 to 1.03)	<0.001	1.03	(1.03 to 1.03)	<0.001
Marital status (ref. not married nor in a civil union)	0.76	(0.68 to 0.83)	<0.001	0.99	(0.93 to 1.06)	0.827
Formal education (years)	0.96	(0.94 to 0.98)	<0.001	0.99	(0.98 to 1.00)	0.126
Religion (ref. no religion or other religion)						
Catholic	1.01	(0.91 to 1.12)	0.889	1.17	(1.04 to 1.32)	0.010
Protestant	0.98	(0.87 to 1.10)	0.687	1.12	(0.99 to 1.27)	0.074
Ethnicity (ref. not indigenous)	1.27	(1.12 to 1.44)	<0.001	1.10	(0.99 to 1.23)	0.086
Food insecurity (ref. has sufficient food)	2.15	(1.99 to 2.33)	<0.001	2.01	(1.89 to 2.13)	<0.001
Household						
Number of household members	1.00	(0.97 to 1.03)	0.993	1.01	(0.99 to 1.03)	0.517
% household members w/depression	1.41	(1.27 to 1.57)	<0.001	1.29	(1.19 to 1.41)	<0.001
Village						
Village population	1.00	(1.00 to 1.00)	0.488	1.00	(1.00 to 1.00)	0.415
Village isolation (# access routes)	1.07	(0.99 to 1.15)	0.083	1.07	(0.99 to 1.14)	0.074
Social network						
Number of friends (degree)	0.99	(0.98 to 1.01)	0.291	0.99	(0.98 to 1.00)	0.227
% friends w/depression	1.39	(1.15 to 1.67)	<0.001	1.38	(1.21 to 1.58)	<0.001
Number of adversaries (degree)	1.08	(1.05 to 1.11)	<0.001	1.09	(1.07 to 1.11)	<0.001
Average degree of friends	1.00	(0.98 to 1.02)	0.984	1.00	(0.99 to 1.02)	0.640
Triadic network characteristics*						
Negative triad (ref. balanced triad)	1.03	(0.94 to 1.13)	0.511	1.03	(0.97 to 1.10)	0.292
Incomplete triad (ref. balanced triad)	1.01	(0.99 to 1.02)	0.608	1.03	(1.01 to 1.04)	<0.001
Village friend network density	1.05	(0.97 to 1.13)	0.275	1.04	(0.97 to 1.11)	0.259
Village adversary network density	1.06	(1.00 to 1.13)	0.069	1.02	(0.97 to 1.08)	0.387
Wave of data collection	0.98	(0.88 to 1.08)	0.625	0.95	(0.88 to 1.02)	0.152

* Triadic network characteristics represent the proportion of friend-pairs among each participant’s friends that form a negative or incomplete triad, respectively. ORs are expressed per 10 percentage-point increase in the proportion of friend-pairs involved in the given triad type.

### Depression covariates

Associations between depression and most demographic and social covariates were qualitatively similar across models (tables 2 and 3). The effect sizes and significance of these individual traits were nearly identical for both models, examining social intransitivity and triadic network characteristics, respectively. For concision and readability, we have reported here the ORs for the covariates in the social intransitivity model while all ORs for the triadic network characteristics model are reported in table 3.

Both men and women were more likely to be depressed if they were older (OR=1.03, 95% CI 1.02 to 1.03 for men; OR=1.03, 95% CI 1.03 to 1.03 for women, for each year of age) and reported experiencing food insecurity (OR=2.16, 95% CI 2.00 to 2.33 for men; OR=1.99, 95% CI 1.87 to 2.11 for women). Participants were more likely to be depressed if a higher proportion of their household members were depressed (OR=1.43, 95% CI 1.28 to 1.58 for men; OR=1.30, 95% CI 1.19 to 1.41 for women) and if more of their friends were depressed (OR=1.30, 95% CI 1.10 to 1.55 for men; OR=1.35, 95% CI 1.20 to 1.52 for women). Additionally, participants with more negative ties had increased odds of depression (OR=1.08, 95% CI 1.05 to 1.11 for men; OR=1.09, 95% CI 1.07 to 1.11 for women).

There were also several factors negatively and positively associated with depression, specific to each gender. Men had decreased odds of depression if they were married or in a civil union (OR=0.75, 95% CI 0.68 to 0.83) or had received more education (OR=0.96, 95% CI 0.94 to 0.98), while they had increased odds of depression if they identified as indigenous (OR=1.25 95% CI 1.10 to 1.41). These covariates were not significant for women. In contrast, women had increased odds of depression if they identified as Catholic (OR=1.17, 95% CI 1.04 to 1.31).

Village-level attributes and structural social measures were largely not significantly associated with the odds of depression for men or women. The number of friends and social isolation of friends were not significantly associated with odds of depression and neither were the densities of positive and negative ties in the village network.

### Postpartum depression symptoms

**Figure 5:**
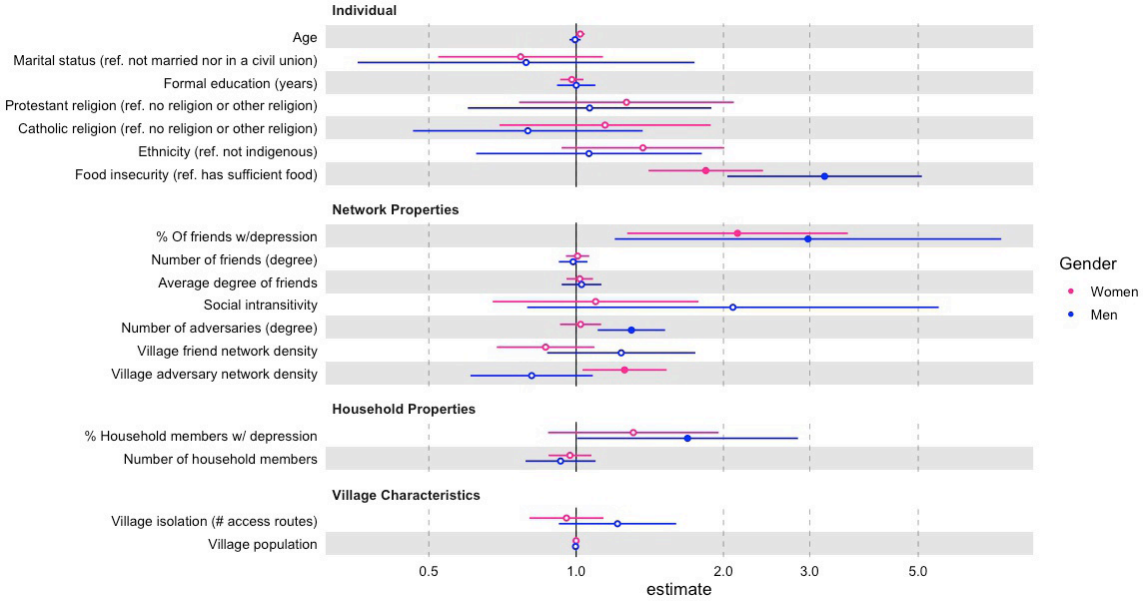
Mixed-effects logistic regression of postpartum depression. Each point represents an OR from the logistic regressions analysing postpartum depression for women (n=1109, conditional R^2^=0.083) and men (n=580, conditional R^2^=0.227). Colour-filled points indicate significant values (p<0.05), and unfilled points (white) indicate non-significant values. The lines extending from each point demarcate the CI. The ORs and CIs are coloured by gender with pink representing values for women and blue for men.

While there were some similarities in factors negatively associated with postpartum depression among men and women, as compared with depression, none of the same positively associated variables were significant (table 4). Figure 5 illustrates the ORs and 95% CIs for each logistic regression estimate. Consistent with our findings among non-recent parents, postpartum fathers and mothers were more likely to be depressed if they were experiencing food insecurity (OR=3.22, 95% CI 2.04 to 5.09 for men; OR=1.84, 95% CI 1.41 to 2.41 for women) and had more depressed friends (OR=2.98, 95% CI 1.20 to 7.39 for men; OR=2.14, 95% CI 1.27 to 3.59 for women). For postpartum fathers, higher odds of depression were associated with having a greater number of depressed household members (OR=1.69 95% CI 1.00 to 2.84) and more adversaries (OR=1.30, 95% CI 1.11 to 1.52). Among postpartum mothers, higher odds of depression were associated with increased density of negative ties in the community (OR=1.26 95% CI 1.03 to 1.53), though density of negative ties was not associated with depression in our other models. Other social network variables, such as the number of friends, the social isolation of friends and the density of positive ties within the village, were not significantly associated with the odds of depression.

**Table 4:** Mixed-effects logistic regression table of individual and network characteristics on postpartum depression

Variable	Postpartum depression among participants					
	Men			Women		
	OR	(95% CI)	P value	OR	(95% CI)	P value
(Intercept)	0.07	(0.01 to 0.39)	0.002	0.16	(0.05 to 0.45)	0.001
Individual						
Age	1.00	(0.97 to 1.02)	0.707	1.02	(1.00 to 1.04)	0.077
Marital status (ref. not married or in a civil union)	0.79	(0.36 to 1.75)	0.560	0.77	(0.52 to 1.14)	0.187
Formal education (years)	1.00	(0.91 to 1.10)	0.996	0.98	(0.93 to 1.04)	0.468
Religion (ref. no religion or other religion)						
Catholic	0.80	(0.46 to 1.37)	0.410	1.15	(0.70 to 1.88)	0.591
Protestant	1.07	(0.60 to 1.89)	0.829	1.27	(0.77 to 2.10)	0.358
Ethnicity (ref. not indigenous)	1.06	(0.63 to 1.81)	0.823	1.37	(0.93 to 2.01)	0.108
Food insecurity (ref. has sufficient food)	3.22	(2.04 to 5.09)	<0.001	1.84	(1.41 to 2.41)	<0.001
Household						
Number of household members	0.93	(0.79 to 1.10)	0.384	0.97	(0.88 to 1.08)	0.572
% household members w/depression	1.69	(1.00 to 2.84)	0.048	1.31	(0.88 to 1.96)	0.189
Village						
Village population	1.00	(0.99 to 1.00)	0.177	1.00	(1.00 to 1.00)	0.793
Village isolation (# access routes)	1.22	(0.92 to 1.60)	0.167	0.96	(0.80 to 1.14)	0.608
Social network						
Number of friends (degree)	0.99	(0.92 to 1.06)	0.684	1.01	(0.95 to 1.06)	0.808
Number of friends w/depression	2.98	(1.20 to 7.39)	0.019	2.14	(1.27 to 3.59)	0.004
Number of adversaries (degree)	1.30	(1.11 to 1.52)	0.001	1.02	(0.93 to 1.13)	0.671
Average degree of friends	1.03	(0.93 to 1.13)	0.594	1.02	(0.96 to 1.08)	0.595
Social intransitivity	2.09	(0.79 to 5.51)	0.135	1.10	(0.68 to 1.78)	0.711
Village friend network density	1.24	(0.87 to 1.75)	0.233	0.87	(0.69 to 1.09)	0.221
Village adversary network density	0.81	(0.61 to 1.08)	0.154	1.26	(1.03 to 1.53)	0.024
Wave of data collection	0.90	(0.48 to 1.69)	0.739	0.98	(0.69 to 1.40)	0.903

### Wave-specific sensitivity analyses

Overall, sensitivity analyses demonstrated the robustness of covariates over time, with minimal disruption to our main analysis across waves of data collection. Sensitivity analyses of wave-specific mixed effects models and pooled analyses showed no change in depression or postpartum depression between Waves 1 and 2 among both men and women, with no improvement in model fit with an added wave of data. A detailed description of the sensitivity analyses and interpretation of results can be found under the headings ‘Data collection across waves’ in the online supplemental material.

## Discussion

We examined the relationship between social network structure and depression in isolated Honduran villages, emphasising infrequently measured network metrics such as triadic motifs and negative ties in signed sociocentric graphs. Our study used uncommon data from a large cohort of 25 605 adults in 176 villages, in a setting considered high-risk for depression. We estimated six models, evaluating separately by gender and accounting for multilevel correlates that included demographics, established factors negatively associated with mental health outcomes, household characteristics, village-level traits and a range of negative and positive social network metrics. While some results are aligned with previous research on depression and postpartum depression, others add to the literature, particularly given our study design that accounted for gender differences when investigating the relationship between social networks and depression and postpartum depression.

More than one-third of our study participants (34.99%) reported symptoms of depression, a higher prevalence than the global 5% and the 12% Latin America averages.^19 63^ However, other studies in rural Honduras have found similar levels.^64 65^ These populations, as in the villages included in our analyses, live in conditions of intermittent access (during the rainy season), absent or unreliable basic public services (electricity, water, sanitation), limited economic opportunities and almost non-existent health infrastructure or mental healthcare.^19 65^ Systemic economic scarcity and food insecurity, along with gender-based violence and lack of sanitary conditions, cumulatively add to the contextual stressors that have been found to affect depression, along with multiple social inequalities.^3647 66^,^71^

Research on the social determinants of depression has shown that depression is associated with multifactorial stressors and mitigators, in which social networks are importantly implicated^1672^,^74^ in addition to broader factors like living in rural areas, resource-limited settings and LMIC.^19 21 75^ However, studies examining the relationship of depression with community-wide social networks, encompassing face-to-face relationships and patterns of social connection, are scarce.^1629 76^,^78^

Here, we provide a set of social network correlates associated with depression by gender, including both positive and negative ties. We provide insights into a novel set of metrics for the study of depression and networks (triadic motifs, after controlling for social network covariates), in the context of a rural LMIC population. Previous research has mostly focused on egocentric designs within high-income countries,^3436 79^,^81^ workplace settings^82^ or adolescent and school social networks.^83 84^

In both genders, a key factor associated with higher odds of depression was food insecurity. Individuals reporting food insecurity had a significant increase in odds of depression with the strongest association among postpartum men (OR=3.22, 95% CI 2.04 to 5.09) and the weakest association among postpartum women (OR=1.84, 95% CI 1.41 to 2.41). This association is consistent with prior work.^85^,^89^

Our study findings show that the household as an immediate social context plays a significant role in increased odds of depression for both genders. Participants with more depressed household cohabitants had greater odds of depression themselves. Mechanisms underlying this association may be multifactorial, resulting from both biological and social influences that might be tied to kinship, social contagion or a combination of both.^90 91^ Previous longitudinal research on social networks and unhappiness has shown that physical proximity affects shared mental health status, particularly for residents of the same household.^76^ Proximity may even foster transmission of mental health states through shared microbiome exposures.^92^,^94^ We observed that individuals with more depressed friends had increased odds of depression. This association has been observed in previous studies.^16^ In our study, as well, participants with more negative ties had increased odds of depression, aligning with previous work on the adverse effects of negative relationships on stress and mental health.^36 37 95^

Research on negative ties and depression has historically focused on dyadic relationships. Our study explores this relationship at a sociocentric level and within triadic network features.^32^ We observed that participants with more negative ties had higher odds of depression.^96^ While studies have previously noted the outsized importance of negative relationships on psychological well-being, even over the benefits of positive relationships,^33^,^3795^ our findings reflect both the importance of direct negative relationships and an attenuation of association between depression and negative relationships among one’s friends. These findings may reflect the difficulty in quantifying the precise dynamics and co-occurring permutations of positive and negative ties among one’s friends or may simply reinforce the connection between direct negative interactions and depression. There is a need to apply the burgeoning research on negative social networks to enrich our understanding of mental health and to consider the role of negative ties beyond direct connections to an individual.^39^,^41^

As in the classic study by Bearman and Moody^31^, in which suicidality among adolescent girls, though not boys, was significantly associated with more intransitive friendships, this key observation holds strikingly true in our study of depression, even within a highly different age group and cultural context. Our results showed that women with friends who were not socially connected to each other (forming an ‘incomplete triad’ and captured by the measure social intransitivity) were associated with increased odds of depression as compared with women whose friends were friends with one another (forming a ‘balanced triad’). The same was not true of men. Our investigations into the role of negative ties among friends reinforced the idea that closure of relationships among friends is important, rather than the positive-negative valence of these relationships. Though one might assume that negative ties are the opposite of positive ones and that increased negativity among one’s friends might relate to a participant’s poorer psychological health, others have suggested that the true opposite of both positive and negative ties is indifference—as in no association at all.^38^

Finally, our study included a focus on postpartum parents. A key feature of our analyses was the ability to study postpartum depression symptoms in both genders in the context of their face-to-face and signed sociocentric networks. Among postpartum parents (580 men and 1109 women), we observed a depression prevalence of 24.31% for men and 31.29% for women, similar to previous estimates for women, though studies on the prevalence for men are scarce.^97^,^99^ Individual-level associations of food insecurity and proportion of friends with depression were akin to the overall associations with depression previously discussed among the non-recent-parent population, though proportion of friends with depression demonstrated larger effect sizes among postpartum parents. As compared with non-postpartum women, the decrease in associations between social network structure (number of adversaries, social intransitivity) and depression of postpartum women may be linked to a greater role of biology in instigating depression symptoms during the postpartum period. Though much research has been conducted individually on the biological and psychosocial influences on postpartum depression, more work is needed to integrate and compare these factors.^52^

Overall, our findings contribute novel perspectives to studying social networks and depression, including postpartum depression, in rural, low-income communities. While the detrimental effects of negative social relationships on depression may be confined to direct ties with participants, we observe a variable association between depression and higher-order structural social network patterns, specifically for triadic structures. The association between social intransitivity and depression, alongside the depression status of household members and friends, indicates the importance of these broader social network structures in mental health and reiterates the potential vulnerable status of women to negative social network effects.^43^ The lack of association between postpartum depression and both social intransitivity and household depression status may point to a stronger biological component and differential social network correlates of postpartum depression.

### Limitations

This study has several limitations. First, it was conducted in a single country (Honduras), and our results may not be generalisable beyond this population. Second, measurement of mental health outcomes in these villages may be constrained by cultural stigma.^100^ Disclosure of mental health symptoms, especially in contexts with limited access to mental health resources may be difficult. Third, because our models are cross-sectional and observational, we do not make causal claims regarding the effects of specific social network structures on depression.

## Conclusion

Prior research has linked social networks with depression.^1634^,^36 66^ Still, few studies have explored structural social network correlates such as triadic motifs and negative tie measures within face-to-face sociocentric networks. Our study offers a new perspective on multilevel factors (household, triads, networks and community characteristics) through gender-specific modelling of depression and postpartum depression in a large sample of individuals residing in a resource-limited context, such as rural Honduras. When it comes to depression, who a person’s friends are, whether they are depressed and how they are connected, all appear to matter.

## Supplementary material

10.1136/bmjopen-2025-108285 online supplemental file 1

## Data Availability

Data are available upon reasonable request.
