# Supplementary material for: Social network structure and depression by gender in rural Honduras: a cross-sectional study
Source: BMJ Open. 2026 Jun 12;16(6):e108285. doi: 10.1136/bmjopen-2025-108285 (PMC13288701; doi:10.1136/bmjopen-2025-108285)
Supplement: online supplemental file 1 [file bmjopen-16-6-s001.docx]

**Supplemental Material**

1. **Participant selection**

The participants included in this study are those who took part in a parent randomized controlled trial (RCT) (1-3) and completed all survey questions of interest for the waves included in this analysis. Those without complete survey data were excluded from this study (less than 5% of those interviewed for the parent RCT). In Figure 1 of the main manuscript, a detailed description of the longitudinal flow of participants is included. By design, the parent trial used census data (not sampling), meaning that, on average, an uncommonly large percentage of residents for each village were surveyed.

1. **Potential sources of bias**

As in any longitudinal study, one source of bias in the analyses can be the loss of participants to follow-up at subsequent waves. In this specific analysis, this loss may relate to dependent variables in two ways: first, very recent postpartum parents might not have been at home at the time of data collection; and second, clinically depressed individuals in critical episodes may have avoided responding to surveyors. However, as described in the protocol (1) and data descriptions of the parent RCT, the study design took several steps to avoid bias and retain the cohort across time, using a culturally and community–centered approach. All surveys were scheduled at participants’ convenience, and they were allowed to decide when to respond. Villagers were visited in their households in all waves. The RCT had full coverage of the village, enabling all residents to participate in the study.

Another potential source of bias in this study may be related to the low levels of mental health literacy and scarcity of resources to support mental health conditions in the study communities. These factors may have prevented participants from identifying their symptoms or led participants to declare symptoms, in the aim of receiving some support for their communities, as surveys often precede the elaboration of specific health programs. Efforts were made on the parent study to avoid these sources of bias such as having a local team of surveyors and mostly gender-matched interviews; a teach-back approach to the consent process, in which the participants demonstrated awareness of the purpose and importance of the study alongside an understanding that no service would be provided as a result of the study, among other community engagement and socialization measures. These limitations (social desirability bias and loss of the extremes of clinically depressed people) are not uncommon in mental health studies, especially in understudied populations. Separate from the measures described in the original research, we believe that the percentage of individuals that could have been lost due to these reasons may have been negligible, particularly as the parent study reached more than 90% of the population for most villages.

1. **Variable description**

**Main outcome measures**

*Depression and Postpartum Depression*

Our dependent variable of depression was determined using a standard two-item depression screen, PHQ-2 (4). The PHQ-2 consists of two items: how often, over the last two weeks, have participants been bothered by 1) little interest or pleasure in doing things and 2) feeling down, depressed, or hopeless? For both questions, possible responses were: 1) not at all, 2) several days, 3) more than half the days, or 4) nearly every day. Responses were coded from zero to three, with zero for ‘not at all’ and three for ‘nearly every day.’ Both responses were summed, resulting in a combined score. If the participant scored two or more, the variable took the value of one, indicating that major depressive disorder is likely. This cut-off point is recommended in Spanish-speaking populations (5). In the case of postpartum depression, respondents who reported having a child younger than six months the postpartum variable was coded as one and zero otherwise.

**Independent variables**

To construct the friendship and adversarial networks, we utilized name generators with specific questions. For the friendship networks, we asked: ‘Who do you spend your free time with?’, ‘Who is your closest friend?’, and ‘Who do you discuss personal matters with?’. For adversarial networks, the question was: ‘Who are the people with whom you do not get along well?’. Based on these responses, undirected graphs were created for each village, where nodes represent individuals and edges denote their connections, friendly or adversarial.

Social intransitivity was measured by examining all possible pairs among an individual's friends and determining the proportion of friends who were not connected to each other. We then decomposed individual‑level social intransitivity into the composition of ties among pairs of a given participant’s friends. For each participant with at least two friends, we classified each pair of his or her friends as friends (i.e., positive closure, forming a ‘balanced triad’), adversaries (i.e., negative closure, forming a ‘negative triad’), or having no tie (i.e., non-closure, forming an ‘incomplete triad’). We then calculated the proportions of friend‑pairs forming negative triads and incomplete triads, respectively, with balanced triads as the implicit reference. For a more illustrative description, see Figure 2 under Methods in the main text.

**Individual-level covariates**

Individual characteristics included age, marital status, formal education, religion, ethnicity, and food insecurity. Age is a continuous variable measured in years. Marital status is a binary variable, coded as "1" for partnered individuals, including those married or in a civil union, with not married or in a civil union (single, widowed, separated) as the reference category. Formal years of education was coded as a continuous variable: 1st to 6th grade is coded as their respective grades (1-6), ‘Some secondary’ as 8, ‘Secondary’ as 11, ‘More than secondary’ as 13, and ‘Have not completed any type of school’ as 0. Religion was coded as a categorical variable coded as Catholic, Protestant, or Other (including Mormon, other religion, or no religion). Food insecurity was coded as a binary variable using an abbreviated two-question measure based on a longer validated scale (6). Individuals who worried about or ran out of food in the three months before survey data collection were identified as experiencing food insecurity. Ethnicity was coded as a binary variable coded as indigenous if respondents self-reported as belonging to Lenca, Maya Chorti, or another indigenous community.

**Household-level covariates**

We included two variables related to household composition. First, the total number of individuals in the household, and second, the proportion of household members who had depression symptoms based on the cutoffs described above.

**Village-level covariates**

Village characteristics in the model included village size, quantifying the number of individuals within the social network graph at each village. We used number of access routes as a proxy for village isolation, which represents a discrete variable that represents a metric of the availability of routes or roads providing access to or within the village.

**Social network covariates**

From the constructed social networks, the degree of each individual—the number of direct connections they have within their network—was computed using the igraph package. The number of friends with depression was determined as the average proportion of an individual’s friends exhibiting depression symptoms, based on predefined cut-offs mentioned above. The average degree of friends was calculated by finding each node’s neighbours and computing the mean degree of these neighbours. Network density, both for friendship and adversarial networks, was calculated by comparing the number of actual edges to the total number of possible edges in the network.

**Table 1. Pseudo-R^2^ values for all logistic regression models for women and men.**

| **Model** | **Theoretical** | | **Delta** | |
| --- | --- | --- | --- | --- |
|  | **Marginal R^2^** | **Conditional R^2^** | **Marginal R^2^** | **Conditional R^2^** |
| **Full depression model** |  |  |  |  |
| Women (N=14,642) | 0.120 | 0.223 | 0.101 | 0.188 |
| Men (N=10,963) | 0.131 | 0.208 | 0.098 | 0.155 |
| **Triadic network characteristics depression model** |  |  |  |  |
| Women (N=14,397) | 0.120 | 0.218 | 0.120 | 0.048 |
| Men (N=10,725) | 0.135 | 0.206 | 0.135 | 0.055 |
| **Postpartum depression model** |  |  |  |  |
| Women (N=1,109) | 0.083 | 0.083 | 0.062 | 0.062 |
| Men (N=580) | 0.219 | 0.227 | 0.185 | 0.192 |

*Notes*: Differences in N between the full depression model and the triadic network characteristics depression model are due to not every participant being part of a triadic network pattern.

1. **Data collection across waves**

We conducted wave-stratified models and formal between-wave comparisons to analyse differences across waves of data collection over time. The unadjusted prevalence of depression and postpartum depression remained relatively steady over time (Table S2).

**Table 2. Unadjusted prevalence of depression (PHQ-2 ≥ 2) by survey wave.**

| **Wave** | **Depression** | | | **Postpartum depression** | | |
| --- | --- | --- | --- | --- | --- | --- |
|  | **n** | **Cases** | **Prevalence, %** | **n** | **Cases** | **Prevalence, %** |
| **Men** |  |  |  |  |  |  |
| Wave 1 (2015–2016) | 10,396 | 2,864 | 27.5 | 436 | 112 | 25.7 |
| Wave 2 (2019) | 6,318 | 1,877 | 29.7 | 157 | 31 | 19.7 |
| **Women** |  |  |  |  |  |  |
| Wave 1 (2015–2016) | 14,185 | 5,754 | 41.4 | 760 | 249 | 32.8 |
| Wave 2 (2019) | 10,967 | 4,675 | 42.6 | 349 | 108 | 30.9 |

We then determined whether adjusted odds of depression differed across waves by comparing the model with and without the data collection wave added as a fixed effect (Table S3). No difference in adjusted odds of depression was observed between Waves 1 and 2 in the full depression model (OR=0.98, 95% CI 0.89-1.07 for men; OR = 1.05, 95% CI 0.98-1.14 for women) or the triadic network characteristics model (OR=0.98, 95% CI 0.88-1.08 for men; OR = 0.95, 95% CI 0.88-1.02 for women). Consistent with this, adding wave as a fixed effect did not improve model fit as compared with the model without wave added in the full depression model (LRT for men: *χ*^2^(2) = 0.29, *p* = 0.592; LRT for women: *χ*^2^(2) = 2.04, *p* = 0.153) or the triadic network characteristics model (LRT for men: χ^2^(2) = 0.27, *p* = 0.603; LRT for women: χ^2^(2) = 2.21, *p* = 0.137), demonstrating no statistically significant change in adjusted odds of depression over time (Table S4).

Using the same test to compare differences in adjusted odds of postpartum depression across waves, we observed no differences in adjusted odds over time for either men or women. There was no statistically significant difference in adjusted odds of postpartum depression between Waves 1 and 2 for men or women (OR=1.11, 95% CI 0*.*59-2.10 for men; OR=1.02, 95% CI 0*.*72-1*.*46 for women). Adding data collection wave as a categorical variable did not improve model fit (LRT for men: *χ*^2^(2) = 0.11, *p* = 0.74; LRT for women: *χ*^2^(2) = 0.02, *p* = 0.90).

**Table 3. Between-wave differences in adjusted odds of depression from the pooled model (Wave 2 is the reference category).**

| **Contrasted timepoints** | **Men** | | | **Women** | | |
| --- | --- | --- | --- | --- | --- | --- |
|  | **OR** | **(95% CI)** | **p** | **OR** | **(95% CI)** | **p** |
| **Full depression model** |  |  |  |  |  |  |
| Wave 1 vs Wave 2 (ref) | 0.976 | (0.886, 1.074) | 0.615 | 1.054 | (0.978, 1.137) | 0.167 |
| **Triadic network characteristics depression model** |  |  |  |  |  |  |
| Wave 1 vs Wave 2 (ref) | 0.976 | (0.884, 1.077) | 0.625 | 0.945 | (0.875, 1.021) | 0.152 |
| **Postpartum depression model** |  |  |  |  |  |  |
| Wave 1 vs Wave 2 (ref) | 1.114 | (0.591, 2.099) | 0.739 | 1.022 | (0.717, 1.458) | 0.903 |

*Notes*: Values represent OR (95% CI) comparing the respective adjusted mixed-effects model when including versus excluding wave as a categorical variable.

**Table 4. Likelihood-ratio test comparing all adjusted pooled logistic regression models, including versus excluding wave as a categorical variable.**

| **Model** | **Men** | | **Women** | |
| --- | --- | --- | --- | --- |
|  | ***χ*^2^(2)** | **p** | ***χ*^2^(2)** | **p** |
| Full depression model | 0.287 | 0.592 | 2.040 | 0.153 |
| Triadic network characteristics depression model | 0.270 | 0.603 | 2.208 | 0.137 |
| Postpartum depression model | 0.111 | 0.739 | 0.015 | 0.903 |

To compare size and significance of estimates for each coefficient over time, we refit each mixed-effects logistic regression models (for depression and postpartum depression) separately within each wave using random intercepts for household and village (Table S5 for depression; Table S6 for depression with triadic network characteristics; Table S7 for postpartum depression). In the wave-stratified models for postpartum men, only a random intercept for village was included due to small sample size resulting in singular fits when adding a random intercept for household.

| **Variable** | **Depression Among Participants** | | | |
| --- | --- | --- | --- | --- |
|  | **Men** | | **Women** | |
|  | **Wave 1**  **(n=10,396)** | **Wave 2**  **(n=6,318)** | **Wave 1**  **(n=14,185)** | **Wave 2**  **(n=10,967)** |
| (Intercept) | **0.079 (0.193)***** | **0.109 (0.193)***** | **0.108 (0.166)***** | **0.140 (0.151)***** |
| **Individual** |  |  |  |  |
| Age | **1.026 (0.002)***** | **1.025 (0.002)***** | **1.028 (0.001)***** | **1.027 (0.002)***** |
| Marital status (ref. not married nor in a civil union) | **0.784 (0.060)***** | **0.710 (0.076)***** | 1.021 (0.040) | 0.961 (0.046) |
| Formal education (years) | **0.965 (0.011)***** | **0.951 (0.013)***** | 0.988 (0.008) | 0.992 (0.009) |
| Religion (ref. no religion or other religion) |  |  |  |  |
| Catholic | 1.001 (0.064) | 1.026 (0.083) | **1.223 (0.073)**** | 1.094 (0.084) |
| Protestant | 0.997 (0.069) | 0.912 (0.090) | 1.156 (0.074) | 1.023 (0.086) |
| Ethnicity (ref. not indigenous) | **1.405 (0.076)***** | 1.007 (0.089) | **1.210 (0.069)**** | 0.930 (0.072) |
| Food insecurity (ref. has sufficient food) | **2.124 (0.049)***** | **2.247 (0.060)***** | **1.846 (0.038)***** | **2.193 (0.043)***** |
| **Household** |  |  |  |  |
| Number of household members | 1.009 (0.015) | 0.972 (0.025) | 1.014 (0.012) | 0.995 (0.019) |
| % household members w/ depression | **1.425 (0.068)***** | **1.431 (0.081)***** | **1.330 (0.056)***** | **1.263 (0.062)***** |
| **Village** |  |  |  |  |
| Village population | 1.000 (0.001) | 1.000 (0.001) | 0.999 (0.001) | 0.999 (0.001) |
| Village isolation (# access routes) | **1.092 (0.044)*** | 1.014 (0.042) | **1.099 (0.041)*** | 1.014 (0.035) |
| **Social network** |  |  |  |  |
| Number of friends (degree) | 0.989 (0.007) | 1.004 (0.012) | 0.994 (0.006) | 0.995 (0.009) |
| % friends w/depression | **1.370 (0.117)**** | 1.201 (0.124) | **1.456 (0.085)***** | **1.237 (0.080)**** |
| Number of adversaries (degree) | **1.079 (0.015)***** | **1.093 (0.037)*** | **1.083 (0.011)***** | **1.118 (0.019)***** |
| Average degree of friends | 0.994 (0.011) | 1.015 (0.014) | 1.004 (0.009) | 1.003 (0.010) |
| Social intransitivity | 1.162 (0.114) | 0.959 (0.103) | 1.146 (0.080) | **1.356 (0.071)***** |
| Village friend network density | 1.002 (0.051) | **1.145 (0.060)*** | 1.002 (0.047) | 1.042 (0.043) |
| Village adversary network density | **1.088 (0.040)*** | 0.978 (0.046) | 0.996 (0.037) | 1.026 (0.036) |

**Table 5. Wave-specific mixed-effects logistic regression models (separate fits by wave) for individual and network characteristics on depression**

*Notes*: Cells report odds ratios; parentheses contain the standard error of the log-odds coefficient (SE_log(OR)_).

Significance: ∗*p <* 0*.*05, ∗∗*p <* 0*.*01, ∗∗∗*p <* 0*.*001.

**Table 6. Wave-specific mixed-effects logistic regression models (separate fits by wave) for individual and triadic network characteristics on depression**

| **Variable** | **Depression Among Participants** | | | |
| --- | --- | --- | --- | --- |
|  | **Men** | | **Women** | |
|  | **Wave 1** | **Wave 2** | **Wave 1** | **Wave 2** |
| (Intercept) | **0.073 (0.214)***** | **0.104 (0.226)***** | **0.110 (0.177)***** | **0.121 (0.173)***** |
| **Individual** |  |  |  |  |
| Age | **1.027 (0.002)***** | **1.026 (0.002)***** | **1.028 (0.001)***** | **1.025 (0.002)***** |
| Marital status (ref. not married nor in a civil union) | **0.771 (0.061)***** | **0.737 (0.080)***** | 1.014 (0.041) | 0.963 (0.049) |
| Formal education (years) | **0.964 (0.011)***** | **0.952 (0.014)***** | 0.989 (0.008) | 0.991 (0.009) |
| Religion (ref. no religion or other religion) |  |  |  |  |
| Catholic | 0.997 (0.065) | 1.031 (0.088) | **1.239 (0.075)**** | 1.078 (0.091) |
| Protestant | 0.994 (0.070) | 0.929 (0.095) | **1.180 (0.076)*** | 1.015 (0.093) |
| Ethnicity (ref. not indigenous) | **1.418 (0.077)***** | 1.026 (0.092) | **1.216 (0.070)**** | 0.895 (0.075) |
| Food insecurity (ref. has sufficient food) | **2.111 (0.050)***** | **2.262 (0.063)***** | **1.833 (0.038)***** | **2.277 (0.045)***** |
| **Household** |  |  |  |  |
| Number of household members | 1.010 (0.016) | 0.980 (0.026) | 1.012 (0.012) | 1.000 (0.019) |
| % household members w/ depression | **1.399 (0.070)***** | **1.420 (0.083)***** | **1.347 (0.057)***** | **1.228 (0.065)**** |
| **Village** |  |  |  |  |
| Village population | 1.000 (0.001) | 1.000 (0.001) | 0.999 (0.000) | 1.000 (0.001) |
| Village isolation (# access routes) | 1.086 (0.044) | 1.013 (0.044) | **1.101 (0.041)*** | 1.011 (0.035) |
| **Social network** |  |  |  |  |
| Number of friends (degree) | 0.989 (0.007) | 1.003 (0.012) | 0.994 (0.006) | 0.995 (0.009) |
| % friends w/depression | **1.405 (0.125)**** | **1.328 (0.141)*** | **1.426 (0.092)***** | **1.310 (0.093)**** |
| Number of adversaries (degree) | **1.078 (0.016)***** | **1.095 (0.038)*** | **1.083 (0.011)***** | **1.112 (0.020)***** |
| Average degree of friends | 0.996 (0.012) | 1.014 (0.016) | 1.001 (0.010) | 1.017 (0.012) |
| Triadic network characteristics^1^ |  |  |  |  |
| Negative triad (ref. balanced triad) | 1.027 (0.058) | 1.047 (0.069) | 1.002 (0.045) | 1.058 (0.042) |
| Incomplete triad (ref. balanced triad) | 1.022 (0.014) | 0.992 (0.013) | 1.011 (0.009) | **1.039 (0.009)***** |
| Village friend network density | 1.007 (0.051) | 1.114 (0.061) | 1.006 (0.047) | 1.047 (0.044) |
| Village adversary network density | **1.092 (0.041)*** | 0.984 (0.048) | 0.995 (0.038) | 1.028 (0.037) |

*Notes*: Cells report odds ratios; parentheses contain the standard error of the log-odds coefficient (SE_log(OR)_).

Significance: ∗*p <* 0*.*05, ∗∗*p <* 0*.*01, ∗∗∗*p <* 0*.*001.

^1^Triadic network characteristics represent the proportion of friend-pairs among each participant’s friends that form a negative or incomplete triad, respectively.

| **Variable** | **Postpartum Depression Among Participants** | | | |
| --- | --- | --- | --- | --- |
|  | **Men** | | **Women** | |
|  | **Wave 1**  **(n=436)** | **Wave 2**  **(n=157)** | **Wave 1**  **(n=760)** | **Wave 2**  **(n=349)** |
| (Intercept) | **0.071 (1.185)∗** | **0.018 (1.927)∗** | **0.108 (0.680)**** | 0.177 (0.898) |
| **Individual** |  |  |  |  |
| Age | 0.986 (0.016) | 1.025 (0.037) | 1.014 (0.013) | **1.046 (0.023)*** |
| Marital status (ref. not married nor in a civil union) | 0.823 (0.723) | 0.642 (0.765) | 1.073 (0.284) | **0.450 (0.344)*** |
| Formal education (years) | 1.018 (0.054) | 0.935 (0.118) | 0.945 (0.035) | 1.027 (0.052) |
| Religion (ref. no religion or other religion) |  |  |  |  |
| Catholic | 0.795 (0.313) | 0.456 (0.820) | 1.213 (0.314) | 1.002 (0.456) |
| Protestant | 1.080 (0.331) | 0.933 (0.818) | 1.159 (0.319) | 1.657 (0.470) |
| Ethnicity (ref. not indigenous) | 1.057 (0.316) | 2.217 (0.700) | 1.478 (0.242) | 1.146 (0.368) |
| Food insecurity (ref. has sufficient food) | **3.347 (0.271)∗∗∗** | 2.699 (0.611) | **1.841 (0.167)***** | **1.769 (0.262)*** |
| **Household** |  |  |  |  |
| Number of household members | 0.958 (0.091) | 0.894 (0.283) | 0.981 (0.060) | 0.920 (0.109) |
| % household members w/ depression | **2.000 (0.305)∗** | 0.584 (0.746) | 1.466 (0.243) | 1.177 (0.416) |
| **Village** |  |  |  |  |
| Village population | 0.997 (0.002) | 1.002 (0.007) | 1.001 (0.001) | 0.997 (0.003) |
| Village isolation (# access routes) | 1.283 (0.160) | 0.916 (0.429) | 1.003 (0.105) | 0.827 (0.192) |
| **Social network** |  |  |  |  |
| Number of friends (degree) | 0.986 (0.037) | 0.996 (0.123) | 0.995 (0.033) | 1.068 (0.059) |
| % friends w/depression | 1.664 (0.573) | **28.814 (1.183)∗∗** | **3.202 (0.344)***** | 1.046 (0.455) |
| Number of adversaries (degree) | **1.232 (0.081)∗** | **2.770 (0.431)∗** | 1.031 (0.055) | 0.992 (0.134) |
| Average degree of friends | 1.025 (0.053) | 1.092 (0.140) | 0.993 (0.039) | 1.070 (0.063) |
| Social intransitivity | 3.219 (0.699) | 0.676 (0.968) | 1.222 (0.333) | 0.866 (0.403) |
| Village friend network density | 1.009 (0.210) | **3.203 (0.537)∗** | 0.998 (0.145) | 0.598 (0.282) |
| Village adversary network density | 0.962 (0.162) | 0.335 (0.630) | 1.206 (0.121) | 1.522 (0.219) |

**Table 7. Wave-specific mixed-effects logistic regression models (separate fits by wave) for individual and network characteristics on postpartum depression**

*Notes*: Cells report odds ratios; parentheses contain the standard error of the log-odds coefficient (SE_log(OR)_).

Significance: ∗*p <* 0*.*05, ∗∗*p <* 0*.*01, ∗∗∗*p <* 0*.*001.

In addition, we conducted wave-specific sensitivity analyses to examine the stability of the estimated coefficients across each wave. We used likelihood-ratio tests to determine whether predictor effects changed between waves by adding one wave x predictor interaction at a time (Table S8). For men, only ethnicity showed statistically significant effect modification for depression. For women, covariates with statistically significant effect modification included food insecurity, proportion of friends with depression, and number of adversaries. We did not observe effect modification among covariates for postpartum depression among men, and for postpartum depression among women, only proportion of friends with depression showed effect modification. In the triadic network characteristics model, we observed statistically significant effect modification in food insecurity and number of adversaries only for women (Table S9).

**Table 8. Wave 1 vs Wave 2 effect modification.** Likelihood-ratio tests (LRT) for adding one wave × predictor interaction at a time. Each row represents the p-value of the comparison between a full model with both Waves 1 and 2 and main effects (including wave) against the same model plus the corresponding interaction, indicated by the interaction variable.

| **Interaction variable** | **Depression model** | | **Postpartum depression model** | |
| --- | --- | --- | --- | --- |
|  | **Men** | **Women** | **Men** | **Women** |
| **Individual** |  |  |  |  |
| Age | 0.963 | 0.730 | 0.186 | 0.770 |
| Marital status (ref. not married nor in a civil union) | 0.472 | 0.453 | 0.964 | 0.104 |
| Formal education (years) | 0.425 | 0.966 | 0.396 | 0.126 |
| Religion (ref. no religion or other religion)^1^ |  |  |  |  |
| Catholic | 0.356 | 0.398 | 0.930 | 0.226 |
| Protestant | 0.356 | 0.398 | 0.930 | 0.226 |
| Ethnicity (ref. not indigenous) | **0.037*** | 0.383 | 0.524 | 0.759 |
| Food insecurity (ref. has sufficient food) | 0.504 | **0.001**** | 0.711 | 0.815 |
| **Household** |  |  |  |  |
| Number of household members | 0.514 | 0.456 | 0.632 | 0.996 |
| % household members w/ depression | 0.593 | 0.131 | 0.462 | 0.177 |
| **Village** |  |  |  |  |
| Village population | 0.610 | 0.941 | 0.576 | 0.794 |
| Village isolation (# access routes) | 0.511 | 0.243 | 0.461 | 0.432 |
| **Social network** |  |  |  |  |
| Number of friends (degree) | 0.447 | 0.624 | 0.500 | 0.438 |
| % friends w/depression | 0.279 | **0.012*** | 0.160 | **0.030*** |
| Number of adversaries (degree) | 0.894 | **0.038*** | 0.110 | 0.919 |
| Average degree of friends | 0.161 | 0.966 | 0.266 | 0.389 |
| Social intransitivity | 0.271 | 0.084 | 0.323 | 0.766 |
| Village friend network density | 0.283 | 0.394 | 0.189 | 0.418 |
| Village adversary network density | 0.808 | 0.221 | 0.671 | 0.964 |

*Notes*: Each cell represents the p-value of the likelihood-ratio test.

Significance: ∗*p <* 0*.*05, ∗∗*p <* 0*.*01, ∗∗∗*p <* 0*.*001.

^1^Religion interactions were tested jointly across factor levels; the joint *p*-value is shown for both “Protestant” and “Catholic” for readability.

**Table 9. Wave 1 vs Wave 2 effect modification for triadic network characteristics depression model.** Likelihood-ratio tests (LRT) for adding one wave × predictor interaction at a time. Each row represents the p-value of the comparison between a full model with both Waves 1 and 2 and main effects (including wave) against the same model plus the corresponding interaction, indicated by the interaction variable.

| **Variable** | **Triadic network characteristics depression model** | |
| --- | --- | --- |
|  | **Men** | **Women** |
| **Individual** |  |  |
| Age | 0.883 | 0.391 |
| Marital status (ref. not married nor in a civil union) | 0.875 | 0.580 |
| Formal education (years) | 0.414 | 0.990 |
| Religion (ref. no religion or other religion)^1^ | 0.456 | 0.306 |
| Catholic |  |  |
| Protestant |  |  |
| Ethnicity (ref. not indigenous) | 0.060 | 0.230 |
| Food insecurity (ref. has sufficient food) | 0.370 | **<0.001***** |
| **Household** |  |  |
| Number of household members | 0.659 | 0.420 |
| % household members w/ depression | 0.899 | 0.093 |
| **Village** |  |  |
| Village population | 0.536 | 0.969 |
| Village isolation (# access routes) | 0.511 | 0.240 |
| **Social network** |  |  |
| Number of friends (degree) | 0.450 | 0.489 |
| % friends w/depression | 0.646 | 0.061 |
| Number of adversaries (degree) | 0.781 | **0.047*** |
| Average degree of friends | 0.153 | 0.364 |
| Triadic network characteristics^2^ |  |  |
| Incomplete triad (ref. balanced triad) | 0.669 | 0.320 |
| Negative triad (ref. balanced triad) | 0.090 | 0.053 |
| Village friend network density | 0.400 | 0.327 |
| Village adversary network density | 0.797 | 0.154 |

*Notes*: Each cell represents the p-value of the likelihood-ratio test.

Significance: ∗*p <* 0*.*05, ∗∗*p <* 0*.*01, ∗∗∗*p <* 0*.*001.

^1^Religion interactions were tested jointly across factor levels; the joint *p*-value is shown for both “Protestant” and “Catholic” for readability.

^2^Triadic network characteristics represent the proportion of friend-pairs among each participant’s friends that form a negative or incomplete triad, respectively.

1. **Assessing collinearity of predictor variables**

Due to the inclusion of multiple interrelated social network measures, we assessed multicollinearity using generalized variance inflation factors (GVIFs) for the fixed effects in each logistic regression model (Table S10 for depression; Table S11 for depression with triadic network characteristics; Table S12 for postpartum depression). All GVIF ^1/(2Df)^ were small across all models—for the models for depression (men: ≤ 1.65; women: ≤ 1.58), depression with triadic network characteristics (men: ≤ 1.66; women: ≤ 1.58), and postpartum depression (men: ≤ 1*.*79; women: ≤ 1*.*77), indicating low collinearity among included predictors. These values indicate that overlap among included social network measures is unlikely to dilute the estimated association for social intransitivity and depression or postpartum depression.

**Table 10. Collinearity assessment for full depression model.**

| **Variable** | **Men** | | | **Women** | | |
| --- | --- | --- | --- | --- | --- | --- |
|  | **GVIF** | **Df** | ***GVIF* ^1^*^/^*^(2^*^Df^* ^)^** | **GVIF** | **Df** | ***GVIF* ^1^*^/^*^(2^*^Df^* ^)^** |
| **Individual** |  |  |  |  |  |  |
| Age | 1.847 | 1 | 1.359 | 1.577 | 1 | 1.256 |
| Marital status (ref. not married nor in a civil union) | 1.567 | 1 | 1.252 | 1.137 | 1 | 1.066 |
| Formal education (years) | 1.493 | 1 | 1.222 | 1.483 | 1 | 1.218 |
| Religion (ref. no religion or other religion) | 1.049 | 2 | 1.012 | 1.031 | 2 | 1.008 |
| Catholic |  |  |  |  |  |  |
| Protestant |  |  |  |  |  |  |
| Ethnicity (ref. not indigenous) | 1.045 | 1 | 1.022 | 1.024 | 1 | 1.012 |
| Food insecurity (ref. has sufficient food) | 1.059 | 1 | 1.029 | 1.050 | 1 | 1.025 |
| **Household** |  |  |  |  |  |  |
| Number of household members | 1.170 | 1 | 1.082 | 1.304 | 1 | 1.142 |
| % household members w/ depression | 1.243 | 1 | 1.115 | 1.221 | 1 | 1.105 |
| **Village** |  |  |  |  |  |  |
| Village population | 2.533 | 1 | 1.592 | 2.492 | 1 | 1.579 |
| Village isolation (# access routes) | 1.046 | 1 | 1.023 | 1.034 | 1 | 1.017 |
| **Social network** |  |  |  |  |  |  |
| Number of friends (degree) | 1.585 | 1 | 1.259 | 1.700 | 1 | 1.304 |
| % friends w/depression | 1.286 | 1 | 1.134 | 1.185 | 1 | 1.088 |
| Number of adversaries (degree) | 1.186 | 1 | 1.089 | 1.186 | 1 | 1.089 |
| Average degree of friends | 1.522 | 1 | 1.234 | 1.548 | 1 | 1.244 |
| Social intransitivity | 1.168 | 1 | 1.081 | 1.210 | 1 | 1.100 |
| Village friend network density | 2.718 | 1 | 1.649 | 2.299 | 1 | 1.516 |
| Village adversary network density | 1.814 | 1 | 1.347 | 1.644 | 1 | 1.282 |
| **Wave of data collection** | 1.686 | 1 | 1.298 | 1.913 | 1 | 1.383 |

Notes: Generalized variance inflation factors (GVIF) for the mixed-effects logistic regression models (Table 2). For multi-df terms (e.g., religion), we report GVIF ^1/(2Df)^ to place GVIFs on a VIF-like scale.

**Table 11. Collinearity assessment for triadic network characteristics depression model.**

| **Variable** | **Men** | | | **Women** | | |
| --- | --- | --- | --- | --- | --- | --- |
|  | **GVIF** | **Df** | ***GVIF* ^1^*^/^*^(2^*^Df^* ^)^** | **GVIF** | **Df** | ***GVIF* ^1^*^/^*^(2^*^Df^* ^)^** |
| **Individual** |  |  |  |  |  |  |
| Age | 1.858 | 1 | 1.363 | 1.592 | 1 | 1.262 |
| Marital status (ref. not married nor in a civil union) | 1.571 | 1 | 1.253 | 1.140 | 1 | 1.068 |
| Formal education (years) | 1.502 | 1 | 1.225 | 1.493 | 1 | 1.222 |
| Religion (ref. no religion or other religion) | 1.048 | 2 | 1.012 | 1.030 | 2 | 1.007 |
| Catholic |  |  |  |  |  |  |
| Protestant |  |  |  |  |  |  |
| Ethnicity (ref. not indigenous) | 1.046 | 1 | 1.023 | 1.026 | 1 | 1.013 |
| Food insecurity (ref. has sufficient food) | 1.060 | 1 | 1.029 | 1.050 | 1 | 1.025 |
| **Household** |  |  |  |  |  |  |
| Number of household members | 1.175 | 1 | 1.084 | 1.298 | 1 | 1.139 |
| % household members w/ depression | 1.234 | 1 | 1.111 | 1.216 | 1 | 1.103 |
| **Village** |  |  |  |  |  |  |
| Village population | 2.559 | 1 | 1.600 | 2.503 | 1 | 1.582 |
| Village isolation (# access routes) | 1.047 | 1 | 1.023 | 1.035 | 1 | 1.017 |
| **Social network** |  |  |  |  |  |  |
| Number of friends (degree) | 1.453 | 1 | 1.205 | 1.516 | 1 | 1.231 |
| % friends w/depression | 1.285 | 1 | 1.133 | 1.184 | 1 | 1.088 |
| Number of adversaries (degree) | 1.184 | 1 | 1.088 | 1.180 | 1 | 1.086 |
| Average degree of friends | 1.542 | 1 | 1.242 | 1.555 | 1 | 1.247 |
| Triadic network characteristics^1^ |  |  |  |  |  |  |
| Negative triad (ref. balanced triad) | 1.050 | 1 | 1.025 | 1.037 | 1 | 1.018 |
| Incomplete triad (ref. balanced triad) | 1.170 | 1 | 1.082 | 1.175 | 1 | 1.084 |
| Village friend network density | 2.755 | 1 | 1.660 | 2.347 | 1 | 1.532 |
| Village adversary network density | 1.849 | 1 | 1.360 | 1.674 | 1 | 1.294 |
| **Wave of data collection** | 1.670 | 1 | 1.292 | 1.882 | 1 | 1.372 |

Notes: Generalized variance inflation factors (GVIF) for the mixed-effects logistic regression models (Table 2). For multi-df terms (e.g., religion), we report GVIF ^1/(2Df)^ to place GVIFs on a VIF-like scale.

^1^Triadic network characteristics represent the proportion of friend-pairs among each participant’s friends that form a negative or incomplete triad, respectively.

**Table 12. Collinearity assessment for postpartum depression model.**

| **Variable** | **Men** | | | **Women** | | |
| --- | --- | --- | --- | --- | --- | --- |
|  | **GVIF** | **Df** | ***GVIF* ^1^*^/^*^(2^*^Df^* ^)^** | **GVIF** | **Df** | ***GVIF* ^1^*^/^*^(2^*^Df^* ^)^** |
| **Individual** |  |  |  |  |  |  |
| Age | 1.203 | 1 | 1.097 | 1.347 | 1 | 1.161 |
| Marital status (ref. not married nor in a civil union) | 1.521 | 1 | 1.233 | 1.362 | 1 | 1.167 |
| Formal education (years) | 1.267 | 1 | 1.125 | 1.254 | 1 | 1.120 |
| Religion (ref. no religion or other religion) | 1.130 | 2 | 1.031 | 1.072 | 2 | 1.018 |
| Catholic |  |  |  |  |  |  |
| Protestant |  |  |  |  |  |  |
| Ethnicity (ref. not indigenous) | 1.134 | 1 | 1.065 | 1.091 | 1 | 1.044 |
| Food insecurity (ref. has sufficient food) | 1.154 | 1 | 1.074 | 1.072 | 1 | 1.036 |
| **Household** |  |  |  |  |  |  |
| Number of household members | 1.094 | 1 | 1.046 | 1.232 | 1 | 1.110 |
| % household members w/ depression | 1.202 | 1 | 1.097 | 1.273 | 1 | 1.128 |
| **Village** |  |  |  |  |  |  |
| Village population | 2.882 | 1 | 1.698 | 2.681 | 1 | 1.638 |
| Village isolation (# access routes) | 1.206 | 1 | 1.098 | 1.124 | 1 | 1.060 |
| **Social network** |  |  |  |  |  |  |
| Number of friends (degree) | 1.678 | 1 | 1.296 | 1.795 | 1 | 1.340 |
| % friends w/depression | 1.222 | 1 | 1.106 | 1.188 | 1 | 1.090 |
| Number of adversaries (degree) | 1.345 | 1 | 1.160 | 1.380 | 1 | 1.175 |
| Average degree of friends | 1.694 | 1 | 1.302 | 1.658 | 1 | 1.287 |
| Social intransitivity | 1.189 | 1 | 1.090 | 1.284 | 1 | 1.133 |
| Village friend network density | 3.212 | 1 | 1.792 | 3.147 | 1 | 1.774 |
| Village adversary network density | 2.137 | 1 | 1.462 | 2.373 | 1 | 1.540 |
| **Wave of data collection** | 1.732 | 1 | 1.316 | 1.590 | 1 | 1.261 |

Notes: Generalized variance inflation factors (GVIF) for the mixed-effects logistic regression models (Table 2). For multi-df terms (e.g., religion), we report GVIF ^1/(2Df)^ to place GVIFs on a VIF-like scale.

To assess whether related social network covariates attenuated the association between our independent variables—social intransitivity and triadic network characteristics—and outcomes of depression or postpartum depression, we re-estimated each mixed-effects logistic regression model after removing all structural network measures most likely to overlap with our independent variables (average degree of friends, village friend network density, and village adversary network density). Odds ratios of the independent variables from the full and reduced models are compared in Table S13.

For men in the reduced network overlap model, the association between social intransitivity and depression remained consistent and nonsignificant, and the association between each of the triadic motifs (negative triad and incomplete triad) and depression remained nonsignificant. For women, the association between social intransitivity and depression was unchanged, remaining statistically significant (full model: OR=1.27, 95% CI 1.14–1.41; reduced model: OR=1.26, 95% CI 1.13–1.40). In addition, the association between negative triads and depression remained nonsignificant, while the association between incomplete triads and depression remained significant (full model: OR=1.03, 95% CI 1.01–1.04; reduced model: OR=1.02, 95% CI 1.01–1.04). For both postpartum men and women, the association between social intransitivity and postpartum depression in the reduced model was stable and nonsignificant.

**Table 13. Robustness of key network estimates to removing overlapping network structure covariates for full depression and postpartum depression models.**

| **Predictor** | **Full model** | | | **Reduced network overlap model** | | |
| --- | --- | --- | --- | --- | --- | --- |
|  | **OR** | **(95% CI)** | ***p*** | **OR** | **(95% CI)** | ***p*** |
| **Full depression model** |  |  |  |  |  |  |
| Men |  |  |  |  |  |  |
| Social intransitivity | 1.056 | (0.908, 1.228) | 0.482 | 1.043 | (0.898, 1.212) | 0.582 |
| Women |  |  |  |  |  |  |
| Social intransitivity | **1.265** | **(1.136, 1.409)** | **<0.001***** | **1.255** | **(1.128, 1.398)** | **<0.001***** |
| **Triadic network characteristics depression model** |  |  |  |  |  |  |
| Men |  |  |  |  |  |  |
| Triadic network characteristics^1^ |  |  |  |  |  |  |
| Negative triad (ref. balanced triad) | 1.030 | (0.943, 1.125) | 0.511 | 1.042 | (0.955, 1.137) | 0.359 |
| Incomplete triad (ref. balanced triad) | 1.005 | (0.986, 1.024) | 0.608 | 1.003 | (0.985, 1.021) | 0.739 |
| Women |  |  |  |  |  |  |
| Triadic network characteristics^1^ |  |  |  |  |  |  |
| Negative triad (ref. balanced triad) | 1.034 | (0.972, 1.100) | 0.292 | 1.037 | (0.975, 1.103) | 0.249 |
| Incomplete triad (ref. balanced triad) | **1.025** | **(1.012, 1.038)** | **<0.001** | **1.023** | **(1.010, 1.036)** | **<0.001** |
| **Postpartum depression model** |  |  |  |  |  |  |
| Men |  |  |  |  |  |  |
| Social intransitivity | 2.091 | (0.794, 5.508) | 0.135 | 1.979 | (0.768, 5.100) | 0.157 |
| Women |  |  |  |  |  |  |
| Social intransitivity | 1.096 | (0.675, 1.779) | 0.711 | 1.071 | (0.666, 1.723) | 0.778 |

*Notes*: ORs and 95% CIs are from mixed-effects logistic regression models fit to the same complete-case analytic sample used in Table 2. Reduced model excludes average degree of friends, village friend network density, and village adversary network density.

^1^Triadic network characteristics represent the proportion of friend-pairs among each participant’s friends that form a negative or incomplete triad, respectively.

As an additional robustness check, we re-estimated each logistic regression model after dropping one network covariate (e.g., density or degree) at a time to confirm whether the estimates for social intransitivity (Table S14) and triadic network characteristics (Table S15) remained unchanged, respectively. Across all models for depression, depression with triadic network characteristics, and postpartum depression, for both men and women, the estimate for our independent variables remained unchanged.

**Table 14. Leave-one-out sensitivity of social intransitivity and adversary degree estimates (dropping one network term at a time) for full depression and postpartum depression models.**

| **Dropped network term** | **Men** | | | **Women** | | |
| --- | --- | --- | --- | --- | --- | --- |
|  | **Social intransitivity OR** | **(95% CI)** | ***p*** | **Social intransitivity OR** | **(95% CI)** | ***p*** |
| **Full depression model** |  |  |  |  |  |  |
| Number of friends (degree) | 1.031 | (0.893, 1.191) | 0.674 | **1.236** | **(1.117, 1.368)** | **<0.001***** |
| % friends w/ depression | 1.050 | (0.903, 1.221) | 0.526 | **1.274** | **(1.144, 1.419)** | **<0.001***** |
| Number of adversaries (degree) | 1.059 | (0.911, 1.232) | 0.454 | **1.289** | **(1.157, 1.435)** | **<0.001***** |
| Average degree of friends | 1.055 | (0.908, 1.227) | 0.483 | **1.264** | **(1.135, 1.407)** | **<0.001***** |
| Village friend network density | 1.050 | (0.903, 1.220) | 0.528 | **1.262** | **(1.133, 1.405)** | **<0.001***** |
| Village adversary network density | 1.057 | (0.909, 1.230) | 0.469 | **1.265** | **(1.135, 1.409)** | **<0.001***** |
| **Postpartum depression model** |  |  |  |  |  |  |
| Number of friends (degree) | 1.997 | (0.776, 5.134) | 0.151 | 1.122 | (0.718, 1.752) | 0.614 |
| % friends w/ depression | 2.084 | (0.794, 5.467) | 0.136 | 1.107 | (0.684, 1.792) | 0.679 |
| Number of adversaries (degree) | 2.085 | (0.795, 5.472) | 0.135 | 1.098 | (0.677, 1.782) | 0.704 |
| Average degree of friends | 2.023 | (0.779, 5.257) | 0.148 | 1.076 | (0.666, 1.737) | 0.765 |
| Village friend network density | 2.039 | (0.778, 5.344) | 0.147 | 1.114 | (0.687, 1.806) | 0.662 |
| Village adversary network density | 2.095 | (0.797, 5.504) | 0.133 | 1.097 | (0.676, 1.778) | 0.708 |

*Notes:* Each row reports the odds ratio (OR) and Wald 95% confidence interval for social intransitivity after refitting the respective mixed effects logistic regression model while dropping the specified network predictor. All other covariates and random effects are unchanged.

**Table 15. Leave-one-out sensitivity of social intransitivity and adversary degree estimates (dropping one network term at a time) for full depression and postpartum depression models.**

| **Dropped network term** | **Negative triad** | | | **Incomplete triad** | | |
| --- | --- | --- | --- | --- | --- | --- |
|  | **OR** | **(95% CI)** | ***p*** | **OR** | **(95% CI)** | ***p*** |
| **Triadic network characteristics depression model** |  |  |  |  |  |  |
| Men |  |  |  |  |  |  |
| Number of friends (degree) | 1.028 | (0.941, 1.123) | 0.536 | 1.002 | (0.985, 1.021) | 0.786 |
| % friends w/ depression | 1.034 | (0.947, 1.129) | 0.451 | 1.003 | (0.985, 1.022) | 0.734 |
| Number of adversaries (degree) | 1.037 | (0.950, 1.132) | 0.414 | 1.006 | (0.987, 1.024) | 0.556 |
| Average degree of friends | 1.03 | (0.943, 1.125) | 0.512 | 1.005 | (0.987, 1.023) | 0.603 |
| Village friend network density | 1.029 | (0.942, 1.124) | 0.527 | 1.004 | (0.986, 1.023) | 0.641 |
| Village adversary network density | 1.039 | (0.952, 1.134) | 0.387 | 1.005 | (0.987, 1.024) | 0.595 |
| Women |  |  |  |  |  |  |
| Number of friends (degree) | 1.032 | (0.970, 1.098) | 0.323 | **1.023** | **(1.010, 1.036)** | **<0.001** |
| % friends w/ depression | 1.038 | (0.975, 1.104) | 0.241 | **1.025** | **(1.012, 1.039)** | **<0.001** |
| Number of adversaries (degree) | 1.037 | (0.975, 1.103) | 0.253 | **1.028** | **(1.014, 1.041)** | **<0.001** |
| Average degree of friends | 1.034 | (0.971, 1.100) | 0.296 | **1.024** | **(1.011, 1.037)** | **<0.001** |
| Village friend network density | 1.033 | (0.971, 1.099) | 0.303 | **1.025** | **(1.011, 1.038)** | **<0.001** |
| Village adversary network density | 1.037 | (0.974, 1.103) | 0.255 | **1.025** | **(1.012, 1.038)** | **<0.001** |

*Notes:* Each row reports the odds ratio (OR) and Wald 95% confidence interval for social intransitivity after refitting the respective mixed effects logistic regression model while dropping the specified network predictor. All other covariates and random effects are unchanged.

1. **Alternative hierarchical participant-level triad specification**

An alternate version of the triadic measure categorized participants by involvement in *one* dominant, mutually exclusive triad category. Each participant was assigned a category in hierarchical order of negative, balanced, incomplete, or no triads (‘none’). We estimated a model with standard covariates, the same as in Table 2, using Waves 1 and 2 of the data. This specification identified whether participants whose friendships formed *either* an incomplete, negative, or no triad differed in odds of depression from participants whose friendships formed balanced triads (with no negative triads), after covariate adjustment.

The triadic measure described in the main manuscript captures the *proportion* of a person’s friend-pairs involved in negative and incomplete triads. Each approach to social intransitivity (our independent variable in Table 2, encompassing the proportion of a person’s non-connected friend-pairs; the triadic measure in the main text in Table 3; and the triadic measure in Table S16) yielded similar results. Nonetheless, it is important to distinguish between these measures. The model with the triadic measure included in the main text (Table 3) is directly comparable to the initial model for social intransitivity (Table 2) but takes the additional step of interweaving signed networks, allowing for decomposition of non-connected friend-pairs (social intransitivity) into negative or absent-tie friend-pairs—people who do not get along and people who did not nominate one another as friends or adversaries, respectively. For example, a participant with one negative friend-pair and many incomplete friend-pairs was classified as involved in a negative triad in the alternate categorical triadic measure (Table S16), whereas the proportional measure captures both the involvement in a negative triad and the greater proportional involvement in incomplete triads.

We hereby present the results of the mutually exclusive triadic measure specified by hierarchical categories (Table S16). Notably, findings are largely consistent with the proportional triadic measure (Table 3). Men did not differ significantly in odds of depression across any triad category relative to the balanced-triad reference: neither negative triads (OR=1.046, 95% CI 0.928–1.165, p=0.118), incomplete triads (OR=1.052, 95% CI 0.918-1.182, p=0.528), nor the ‘none’ category (OR=0.946, 95% CI 0.779-1.149, p=0.574) were associated with depression. Among women, by contrast, classification into the incomplete triad category was associated with modestly higher odds of depression relative to balanced triads (OR=1.052, 95% CI 1.006-1.098, p=0.003), whereas negative triads were not significantly associated with depression (OR=1.071, 95% CI 0.946-1.196, p=0.141). These results reinforce the robustness of our central findings: that non-closure or disconnection among women’s friends is associated with depression, regardless of model specification. When parsing intransitivity into conflict or lack of connection among a person’s friends—whether using a proportional or mutually exclusive categorical triadic measures—our conclusions remained the same.

**Table 16. Alternative hierarchical participant-level triad specification for depression, adjusted for the same standard covariates as Table 2.**

| **Variable** | **Men** | | | **Women** | | |
| --- | --- | --- | --- | --- | --- | --- |
|  | **OR** | **(95% CI)** | **p-value** | **OR** | **(95% CI)** | **p-value** |
| (Intercept) | **0.091** | **(0.068, 0.122)** | **<0.001** | **0.124** | **(0.096, 0.161)** | **<0.001** |
| **Individual** |  |  |  |  |  |  |
| Age | **1.026** | **(1.023, 1.029)** | **<0.001** | **1.028** | **(1.026, 1.030)** | **<0.001** |
| Marital status (ref. not married nor in a civil union) | **0.753** | **(0.684, 0.830)** | **<0.001** | 0.997 | (0.935, 1.063) | 0.920 |
| Formal education (years) | **0.960** | **(0.944, 0.977)** | **<0.001** | 0.989 | (0.977, 1.002) | 0.088 |
| Religion (ref. no religion or other religion) |  |  |  |  |  |  |
| Catholic | 1.009 | (0.909, 1.120) | 0.869 | 1.174 | **(1.045, 1.318)** | **0.007** |
| Protestant | 0.969 | (0.866, 1.084) | 0.583 | 1.109 | (0.986, 1.248) | 0.085 |
| Ethnicity (ref. not indigenous) | **1.244** | **(1.100, 1.408)** | **<0.001** | 1.115 | (0.999, 1.244) | 0.052 |
| Food insecurity (ref. has sufficient food) | **2.156** | **(1.996, 2.328)** | **<0.001** | **1.986** | **(1.873, 2.106)** | **<0.001** |
| **Household** |  |  |  |  |  |  |
| Number of household members | 0.995 | (0.969, 1.022) | 0.698 | 1.003 | (0.982, 1.025) | 0.770 |
| % household members w/ depression | **1.425** | **(1.283, 1.582)** | **<0.001** | **1.298** | **(1.192, 1.414)** | **<0.001** |
| **Village** |  |  |  |  |  |  |
| Village population | 1.000 | (0.999, 1.001) | 0.490 | 1.000 | (0.999, 1.000) | 0.401 |
| Village isolation (# access routes) | 1.071 | (0.996, 1.151) | 0.064 | 1.064 | (0.993, 1.140) | 0.080 |
| **Social network** |  |  |  |  |  |  |
| Number of friends (degree) | 0.995 | (0.981, 1.008) | 0.429 | 0.999 | (0.988, 1.011) | 0.911 |
| % friends w/depression | **1.301** | **(1.097, 1.543)** | **0.002** | **1.350** | **(1.199, 1.520)** | **<0.001** |
| Number of adversaries (degree) | **1.082** | **(1.051, 1.113)** | **<0.001** | **1.093** | **(1.071, 1.115)** | **<0.001** |
| Average degree of friends | 0.999 | (0.981, 1.016) | 0.889 | 0.998 | (0.984, 1.011) | 0.729 |
| Triadic network characteristics¹ |  |  |  |  |  |  |
| Negative triad (ref. balanced triad) | 1.046 | (0.928, 1.165) | 0.118 | 1.071 | (0.946, 1.196) | 0.141 |
| Incomplete triad (ref. balanced triad) | 1.052 | (0.918, 1.182) | 0.528 | **1.052** | **(1.006, 1.098)** | **0.003** |
| None (ref. balanced triad) | 0.946 | (0.779, 1.149) | 0.574 | 0.877 | (0.767, 1.003) | 0.056 |
| Village friend network density | 1.050 | (0.971, 1.135) | 0.224 | 1.036 | (0.970, 1.106) | 0.290 |
| Village adversary network density | 1.057 | (0.994, 1.123) | 0.076 | 1.019 | (0.967, 1.074) | 0.483 |
| **Wave of data collection** | 1.019 | (0.925, 1.122) | 0.702 | 1.042 | (0.966, 1.123) | 0.287 |

*Notes:* ORs and 95% CIs are from mixed-effects logistic regression models fit to the same two-wave analytic sample used in the main manuscript, with adjustment for standard covariates (same as Table 2). Under this specification, each participant is assigned to one triad category under the hierarchy of negative > balanced > incomplete > no triad (‘none’). These odds ratios compare each person’s dominant triadic categories to the reference of an individual with balanced triads and no negative triads. P-values are from Wald tests.

**References**

1. Shakya HB, Stafford D, Hughes DA, Keegan T, Negron R, Broome J, et al. Exploiting social influence to magnify population-level behaviour change in maternal and child health: study protocol for a randomised controlled trial of network targeting algorithms in rural Honduras. BMJ open. 2017;7(3):e012996.

2. Airoldi EM, Christakis NA. Induction of social contagion for diverse outcomes in structured experiments in isolated villages. Science. 2024;384(6695):eadi5147.

3. Oles W, Alexander M, Negron R, Nelson J, Iriarte E, Airoldi EM, et al. Maternal and child health intervention to promote behaviour change: a population-level cluster-randomised controlled trial in Honduras. BMJ open. 2024;14(6):e060784.

4. Kroenke K, Spitzer RL, Williams JB. The Patient Health Questionnaire-2: validity of a two-item depression screener. Medical care. 2003:1284-92.

5. Martinez A, Teklu SM, Tahir P, Garcia ME. Validity of the Spanish-language patient health questionnaires 2 and 9: a systematic review and meta-analysis. JAMA Network Open. 2023;6(10):e2336529-e.

6. Kumar S, Christakis NA, Pérez-Escamilla R. Household food insecurity and health in a high-migration area in rural Honduras. SSM-Population Health. 2021;15:100885.
