# Supplementary material for: Social network structure and depression by gender in rural Honduras: a cross-sectional study
Source: BMJ Open. 2026 Jun 12;16(6):e108285. doi: 10.1136/bmjopen-2025-108285 (PMC13288701; doi:10.1136/bmjopen-2025-108285)
Supplement: Supplementary file 2 [file bmjopen-2025-108285.reviewer_comments.pdf]

PEER REVIEW HISTORY

BMJ Open publishes all reviews undertaken for accepted manuscripts. Reviewers are asked to complete a checklist review form and are provided with free text boxes to elaborate on their assessment. These free text comments are reproduced below.

ARTICLE DETAILS

Title (Provisional)

Social network structure and depression by gender in rural Honduras: a cross-sectional study

Authors

Lee, Selena T.; Papamichalis, Marios; Raygoza-Cortez, Karina; Christakis, Nicholas; Rodriguez de la Rosa, Ana Lucia

---

VERSION 1 - REVIEW

Reviewer 1

Name Gangadharan, Satheesh

Affiliation Loughborough University Loughborough School of Design and Creative Arts, Design school

Date 23-Sep-2025

COI None

This is an excellent article focusing on an important area. The relationship between depression and nature of the social network has been a focus of research for some time. However the stregth of this work is focusing on infrequently measured metrics as well as taking a socio-centric view.

The data on males and females being analysed seperately and having access to a reasonably large number of data were the strengths of the study. A number of weakness have been highlighted apropriately by authors as well.

The paper is written well in clear language.

However I would suggest the following

1. Some description of how the data was collected in the three waves (given the time difference), some methodologies may change. It will be useful to know this. If there were changes, likely impact on the findings as data was pooled in the analysis here.

2. I am interested in knowing if a seperate anlaysis of the data from each wave is possible to see if there are significant differences. Particularly between wave 2 and 3, covid pandemic

took place and the third wave was straight after or even starting from the tail end of covid pandemic.

I have no other comments.

2.

**Reviewer**	**2**
**Name**	**Gizaw, Muluken**
**Affiliation**	**Addis Ababa University, Department of Epidemiology and Biostatistics**
**Date**	**14-Oct-2025**
**COI**	**None**

I have mainly looked on the methods sections, it seems the research was well designed and I have not get a major problem on the designing and excuting the research. However, I was not very clea

**Reviewer**	**3**
**Name**	**Dack, Kyle**
**Affiliation**	**University of Bristol, MRC Integrative Epidemiology Unit**
**Date**	**23-Nov-2025**
**COI**	**None**

Statistical review summary:

The methodology and statistical analysis are reasonable and results were interpreted within the limits of the data. Overall the paper is very well written and addresses an interesting research question.

I have left detailed feedback below. Most concerns the modelling strategy – some elements would benefit from being clarified, particularly a stronger distinction between exposures of interest and covariates used to minimize confounding.

Abstract:

1. The abstract is clear and complete. My only suggestion is to avoid “increased risk” to report odds ratios. This term implies the exposure occurred before the outcome, which is not determined in this cross-sectional data and risk was not modelled.

Methods:

2. The authors reference a prior RCT and protocol as containing further details of the methods relevant to this study, but to ensure alignment with STROBE, a minimum of information should also be provided in this manuscript so that readers understand the context of the study. In particular a brief summary of the study setting, recruitment and survey procedure, and the primary inclusion/exclusion criteria. I suggest adding details of these under a heading or headings after the "study sample" heading.

3. Independent/explanatory variables: Based on the introduction and statistical analysis, I think that the independent variable of interest is intransitive friendships.

-However, the methods describe this variable under "social network construction", and this is followed by a separate "explanatory variables and covariates" section. This implies there are other variables of interest, but the section does not explain which variables are explanatory and which are covariates. This is important to clarify so that readers understand which variables are to be interpreted, and what steps were taken to minimize confounding bias.

-I recommend adding statements to clarify which variables are the IV and which are covariates, and aligning the headings for these sections to avoid any misunderstanding.

4. "All models were stratified by gender" I suggest clarifying that this means they were modelled separately.

5. A random intercept is included to account for potential repeated measures across waves. To help readers interpret this, it may be beneficial to clarify that this means the model reflects the average association across all waves.

6. Given that individuals may have multiple measurements from different waves, which measurement was used to generate descriptive statistics (eg appendix table 1)? I recommend adding a footnote to this table to clarify the approach, e.g. if it takes the most recent wave data for each individual.

Results:

7. To ensure a logical flow of results, Appendix Table 1 (descriptive characteristics of the sample) should be referenced and described prior to figure 3 (model results).

8. Model results: There are a large number of significant findings. It would help readers to interpret these by first highlighting the results most relevant to the study aim, which I assume are the model results related to social network status. Currently network properties are not really reported in-text but these were stated as the main aim. Reporting covariate associations is reasonable but less important given that these may have a different set of confounding variables and cannot be interpreted strongly (i.e. risk of table 2 fallacy).

-Similar to my suggestion for the methods, I recommend signposting the main model results of interest more directly, and ensuring readers understand which variables are only covariates.

9. Appendix tables 1-3. Given the importance of these to the results, I recommend the authors consider if they could be included in the main paper rather than only as an appendix. I believe BMJ Open does allow for multiple tables.

10. Appendix table 2. I was unsure why the title label refers to "depression outcomes" (plural) rather than depression? In-text it is referred to as odds of depression.

11. Appendix table 2. A number of the social network items overlap in terms of what is being measured. Was collinearity assessed, and did the authors consider sensitivity analyses where some of these are removed? It may be that for example, the association between social intransitivity and the outcome is being diluted as some of the effect is captured by other variables.

12. "incidence of depression" is not suitable to describe the odds ratio as there was no incidence measure – other terms used in the results would be more accurate.

13. Appendix table 3. What was the rationale for this model not including covariates to minimize confounding bias? If there are assumed to be no such factors I recommend stating it in the methods to make it clear this risk was evaluated.

Discussion

14. The results are interpreted clearly and reasonably, and limitations/caveats are acknowledged well. I do not have any major comments regarding the discussion.

15. "Individuals reporting food insecurity had an 85–222% increase in odds of depression or postpartum depression". I could not locate which statistics this was based upon. For postpartum depression the 95% CI were 2.04-5.09 for men, 1.44-2.37 for women. For all depression, it was 2.03-2.35 for men and 1.90-2.11 for women.

**Reviewer**	**4**
**Name**	**Hashimoto, Kenji**
**Affiliation**	**Chiba University Center for Forensic Mental Health**
**Date**	**20-Jan-2026**
**COI**	**None**

In this study, the authors examined the association between individuals' structural positions within their village social networks and symptoms of depression and postpartum depression among both men and women. This was a community-based cross-sectional study conducted in 176 villages in the Copán region of Honduras. The participants were village residents, comprising 27,183 adults sampled from data collected between October 2015 and July 2023. The main outcomes were the prevalence of depression and postpartum depression among men and women. Across all participants, 34.97% reported depressive symptoms (40.44%

among women and 27.62% among men). Among recent parents with a new child in the past six months, 30.16% reported postpartum depressive symptoms (33.00% among women and 24.31% among men). Women in intransitive friendship structures (i.e., when their friends are not friends with each other) had an increased risk of depressive symptoms (OR = 1.18, 95% CI 1.07–1.30), an association not observed in men or in postpartum parents. In addition, a higher prevalence of depressive symptoms was found among individuals whose friends had negative social relationships with each other ("negative triads") in both men (OR = 1.11, 95% CI 1.00–1.23) and women (OR = 1.21, 95% CI 1.13–1.30). These findings highlight the importance of examining structural properties of social networks beyond simple dyadic ties, as well as the relevance of including adversarial ties when studying the links between psychological health and face-to-face relationships in resource-limited settings. The findings of this study are interesting, and the manuscript is well written. I have two minor comments:

1) In this study, 34.97% of participants reported depressive symptoms, suggesting a higher prevalence compared with many other countries. Could socioeconomic factors (e.g., low income or lack of access to smartphones and digital communication) contribute to this higher prevalence?

2) The data were collected between October 2015 and July 2023. Did the COVID-19 pandemic influence the prevalence of depressive symptoms in this population?

VERSION 1 - AUTHOR RESPONSE

Reviewer 1 Prof. Satheesh Gangadharan

1. This is an excellent article focusing on an important area. The relationship between depression and nature of the social network has been a focus of research for some time. However the strength of this work is focusing on infrequently measured metrics as well as taking a socio-centric view. The data on males and females being analysed seperately and having access to a reasonably large number of data were the strengths of the study. A number of weakness have been highlighted apropriately by authors as well. The paper is written well written and clear. However, I would suggest the following: Some description of how the data was collected in the three waves (given the time difference), some methodologies may change. It will be useful to know this. If there were changes, the likely impact on the findings as data the data in the analysis here. Provide a description of how the data were collected across three waves.

Response: We appreciate this comment and the overall evaluation of our work. We have added a dedicated subsection titled "Data collection across waves" in the Supplemental Material (pg. 3-9) and a summary in the methods (pg. 8) and results sections (pg. 21-22). The methods section now states that we made no changes to the data collection process, except for excluding two villages due to security concerns during data collection. We have specified the instrument consistency, field protocols, and procedural adherence across waves in the methods section (pg. 8). We also discuss potential implications for pooled models (pg. 21-22)

and ran separate analyses by wave and sensitivity checks between waves, available in the Supplemental Material (pg. 3-9).

2. Concerning the timing of COVID-19 between Waves 3 and 4.

Response: We thank you for your comment. Other reviewers also raised concerns about potential historical threats and methodological changes in Wave 4 of the parent RCT (fewer villages, no information on men in postpartum parents, different survey instruments). For clarity and methodological consistency in our results, per your and another reviewer's perspectives, we've decided to remove Wave 3 of our analysis (referred to as 'Wave 4' in the parent RCT) to avoid introducing methodological, sample change, or historical effects. In addition to the potential changes the pandemic might have introduced (which, in our evaluation, were minimal), the study population of the parent RCT was downsized from 176 villages to 82 in Wave 3 for exogenous reasons unrelated to this study. Furthermore, the instrument (survey design) was modified, and men were no longer asked about childbearing, among other modifications related to a new parent study. Though COVID case rates appeared low in the subset of the population measured within the parent RCT (only 4.5% of respondents reported having COVID between Waves 2 and 3, with only 46% of those cases diagnosed by a medical professional) and in the study region per a national ecological analysis of COVID cases (1), we cannot rule out effects or confirm the direction of the effects of the pandemic restrictions, as well as ecological effects of major hurricanes (Eta and Iota) that severely damaged the country at the end of 2020.

As the primary objective of this paper is to examine the association between social network structure and depression under relatively stable conditions, and to include postpartum men and women, we believe that objective is best achieved by limiting the sample to homogeneous instruments and sample, and by using full information for all models, given that the additional wave was post-measurement, with a different purpose than the RCT parent study (2, 3). Evaluating whether the global 2020 COVID pandemic, through mechanisms such as lockdown-induced isolation or post-pandemic social reorganization, affected this association would require a distinct empirical design and research question beyond the scope of the present study.

We hope these clarifications are in line with all reviewers' suggestions, as we also provide additional robustness checks for the key models in the main text. We express our gratitude for this revision, which has contributed to improving the clarity and methodological consistency of our work.

Reviewer 2: Dr. Muluken Gizaw, Addis Ababa University, Global Health Group

I have mainly looked at the methods sections, It seems the research was well designed, and I have not had a major problem on the designing and executing the research. However, I was not very clear ****Comment from the Editor: Please note that we contacted the reviewer about their incomplete report. However, they never responded to us. Please respond to their comments as much as possible.*

Response: We thank the reviewer for noting that the design appeared robust.

To improve clarity, we expanded and reordered the Methods sections (adding: 'Study design,' 'Recruitment and study sample,' 'Data source,' 'Independent variables: transitivity, intransitivity, and negative triads,' 'Explanatory variables and covariates') to align with other reviewers' comments (pg. 7-12).

Reviewer 3: Dr. Kyle Dack, University of Bristol

Statistical review summary: The methodology and statistical analysis are reasonable and results were interpreted within the limits of the data. Overall the paper is very well written and addresses an interesting research question.
I have left detailed feedback below. Most concerns the modelling strategy – some elements would benefit from being clarified, particularly a stronger distinction between exposures of interest and covariates used to minimize confounding.

Response: Thank you for your revision. We appreciate the overall impressions of our paper. We have addressed all your comments to clarify and specify the covariates in each of the main models. We believe this version includes a stronger, more robust methods section.

To improve clarity, we expanded and reordered the Methods sections, adding sections like 'Study design,' 'Recruitment and study sample,' 'Data source,' 'Independent variables: transitivity, intransitivity, and negative triads,' and 'Explanatory variables and covariates' to address reviewer comments (pg. 7-12). We also added sensitivity analyses to the Supplemental Material to verify robustness over time and assess multicollinearity, especially among social network variables (pg. 3-15).

1. The abstract is clear and complete. My only suggestion is to avoid reporting "increased risk" when reporting odds ratios. This term implies the exposure occurred before the outcome, which is not determined in this cross-sectional data, and risk was not modelled.

Response: We have replaced all uses of ‘risk’ and ‘incidence’ with terminology appropriate for cross-sectional odds ratios (e.g., ‘higher odds’, ‘decreased odds’, or ‘proportion of participants’).

2. The authors reference a prior RCT and protocol as containing further details of the methods relevant to this study, but to ensure alignment with STROBE, a minimum of information should also be provided in this manuscript so that readers understand the context of the study. In particular, a brief summary of the study setting, recruitment and survey procedure, and the primary inclusion/exclusion criteria. I suggest adding details of these under a heading or set of headings following the “study sample” heading.

Response: We appreciate the suggestion and have added a concise summary under new subsections following ‘Study design’ (renamed from ‘Study sample’), ensuring the manuscript is self-contained (pg. 7-8, new and revised sections—‘Study design’, ‘Recruitment and study sample’, and ‘Data source’).

3. Independent/explanatory variables: Based on the introduction and statistical analysis, I think that the independent variable of interest is intransitive friendships. However, the methods describe this variable under “social network construction”, and this is followed by a separate “explanatory variables and covariates” section. This implies there are other variables of interest, but the section does not explain which variables are explanatory and which are covariates. This is important to clarify so that readers understand which variables are to be interpreted, and what steps were taken to minimize confounding bias. I recommend adding statements to clarify which variables are the IV and which are covariates, and aligning the headings for these sections to avoid any misunderstanding.

Response: We have edited the manuscript to identify intransitive friendships and types of triads (negative, incomplete, balanced) as the primary variables of interest. All other measures, including demographics, village, household, and network variables, are now in the covariates section. Headings have been aligned accordingly (pg. 9-12). The Supplemental Material also includes a detailed description of the coding, which is aligned with this organization. We appreciate the suggestions, and we believe the manuscript is now more readable and clearer.

4. “All models were stratified by gender.” I suggest clarifying that this means they were modelled separately.

Response: This is now clearly stated in the manuscript as follows: “All models were estimated separately for women and men” (pg. 12).

5. A random intercept is included to account for potential repeated measures across waves. To help readers interpret this, it may be beneficial to clarify that the model reflects the average association across all waves.

Response: We explain that the random intercept accounts for repeated measures at the individual level, meaning that the coefficients represent the average association across waves (pg. 12). We have added this to this manuscript for clarity purposes. Thank you.

6. Given that individuals may have multiple measurements from different waves, which measurement was used to generate descriptive statistics (e.g., Appendix Table 1)? I recommend adding a footnote to this table to clarify the approach, e.g. if it takes the most recent wave data for each individual.

Response: We appreciate the recommendation and have added a footnote indicating the descriptive data source (e.g., the baseline demographic information collected in the impanelling survey wave for each participant) to the corresponding demographics table (pg. 15).

7. To ensure a logical flow of results, Appendix Table 1 (descriptive characteristics of the sample) should be referenced and described prior to Figure 3 (model results).

Response: For improved clarity, the description of the distribution of demographic characteristics and reference to Table 1 (now in the main text, not the Supplemental Material) have been moved from Methods to Results (pg. 14-15) ensuring that it is referenced prior to the model results. We appreciate this suggestion for enhanced readability.

8. Model results: There are a large number of significant findings. It would help readers to interpret these by first highlighting the results most relevant to the study aim, which I assume are the model results related to social network status. Currently network properties are not really reported in-text but these were stated as the main aim. Reporting covariate associations is reasonable but less important given that these may have a different set of confounding variables and cannot be interpreted strongly (i.e. risk of table 2 fallacy). Similar to my suggestion for the methods, I recommend signposting the main model results of interest more directly, and ensuring readers understand which variables are only covariates.

Response: We have incorporated these changes and have reorganized the Results section to present the social network variables before the covariates (pg. 15-22, subsections 'Depression and associations with intransitivity and negative triads' and 'Depression covariates'), following your recommendation on highlighting the main results, per our aims. We have also added information from the Supplemental Material tables into the main paper. We believe this improved the paper's readability and clarified the main purpose of our analyses.

9. Appendix tables 1-3. Given the importance of these to the results, I recommend the authors consider if they could be included in the main paper rather than only as an appendix. I believe BMJ Open does allow for multiple tables

Response: Thank you for this suggestion. We have integrated each of these tables (previously Tables 1-3 in the Appendix) into the main text, as well as Table 4 reporting results on postpartum depression for consistency (pg. 15, 17, 18, 21).

10. Appendix table 2. I was unsure why the title label refers to "depression outcomes" (plural) rather than depression. In-text, it is referred to as the odds of depression.

Response: We have standardized terminology to reflect 'depression' rather than 'depression outcomes' throughout the main text, figures, tables, and Supplemental Material. We also clarified in text that depression symptoms are abbreviated as 'depression' for readability purposes (pg. 9).

11. Appendix table 2. A number of the social network items overlap in terms of what is being measured. Was collinearity assessed, and did the authors consider sensitivity analyses where some of these are removed? It may be that for example, the association between social intransitivity and the outcome is being diluted as some of the effect is captured by other variables.

Response: We appreciate the opportunity to expand on this topic. We were careful to introduce social network measures that were not highly correlated or that measured the same type of trait (e.g., degree or eigenvector centrality). We assessed multicollinearity among the social network measures using generalized variance inflation factors (GVIFs) for the fixed effects in each mixed-effects logistic regression model. All $GVIF^{1/(2Df)}$ values were small both for models of depression (men: ≤ 1.65; women: ≤ 1.58) and of postpartum depression (men: ≤ 1.79; women: ≤ 1.77), indicating low collinearity among included predictors. In addition, we conducted sensitivity analyses to address potential overlap among our independent variables and structural social network measures by comparing our standard models to (1) 'reduced overlap models' that dropped potentially redundant structural network terms (average degree of friends, village friendship density, and village adversary density) and (2) leave-one-out refit models, dropping one network covariate at a time. Across these specifications, the social intransitivity estimate remained unchanged and stable across leave-one-out refit models for men and women, indicating low collinearity. These sensitivity analyses have been added to Supplemental Material (pg. 10-15) and the main text in a brief discussion in the Methods (pg. 11-12).

12. "incidence of depression" is not suitable to describe the odds ratio, as there was no incidence measure; other terms used in the results would be more accurate.

Response: We appreciate your suggestion to improve our health terminology precision. We have edited the manuscript to use more consistent in wording, directly referring to the odds of depression when appropriate and to the proportion of depressed participants, or women, when applicable.

13. Appendix table 3. What was the rationale for this model not including covariates to minimize confounding bias? If there are assumed to be no such factors I recommend stating it in the methods to make it clear this risk was evaluated.

Response: Thank you for this comment. This table is now part of the main manuscript (Table 3), and we have made changes to improve clarity and consistency of this model. We believe these modifications have strengthened the study's consistency, unified the aims of our study, and improved the manuscript's readability by retaining in the main text only the models with comparable main variables: transitivity and an adjusted version of triadic motifs (parametrized as proportions) with all covariates. As we will detail bellow, we left alternative specifications for the Supplemental Material.

Specifically, in revising the models in the main manuscript, we made three changes. First, we adjusted all models to make them comparable, including the same standard covariates (as suggested) to match the intransitivity model (Table 2). Second, we re-parametrized the triadic predictor so that it is now defined on the same denominator as social intransitivity: the type of tie among the unordered pairs among a participant's friends. Specifically, for each participant with at least two friends, we still classify each pair of friends as positively connected (transitive friend-pair or 'balanced triad'), negatively connected (adversarial friend-pair or 'negative triad'), or unconnected (intransitive friend-pair or 'incomplete triad'). However, we now model the proportions of negative and unconnected friend-pairs, with positively connected friend-pairs as the reference category, to account for the full set of structures for each participant. The former adjusted triadic model has now been added to the Supplemental Material, as it continues to serve as a sensitivity check. In simple terms, the old version of Table 3 assigned individuals to a mutually exclusive category (or bin) based on whether they belonged to a negative, balanced, incomplete, or null triad, in that hierarchical order. The earlier specification analysed a participant-level categorical association; the revised specification now shows how a participant's friend-pairs are distributed across triad types. Under this parametrized specification, the continuity between the two models (analysing social intransitivity and triadic characteristics, respectively) is clearer.

By including this updated parameterization in the main manuscript, the models now better reflect one of our main contributions: the inclusion of signed ties and triadic motifs. This parametrized measure is not only more sensitive to the mixture of triad types in which individuals are embedded, but it also allows a direct decomposition of social intransitivity (first model) into the effects of disconnection among friends versus conflict among friends, one of the key features of our study.

The hierarchical, mutually exclusive person-level triad classification has been moved to the Supplemental Material (also reported below as Table S16), along with the rest of the newly added sensitivity and robustness analyses.

Lastly, the sum of these changes (adjusting for covariates and refining our triadic measure) resulted in a modification of one of our findings: the association between depression and negative triads is now non-significant. However, the association between depression and incomplete triads among women remained significant, further confirming one

of the core findings of the study and reinforcing the idea that closure of relationships among friends affects depressive symptoms, not the negative valence of these relationships.

We hope these improvements have addressed your questions. This version strengthens the robustness of the measures and models, enhances the clarity of the manuscript results sections, and highlights the main findings across models. We appreciate your contributions to our revisions.

Supplemental Table 16. Alternative hierarchical participant-level triad specification for depression, adjusted for the same standard covariates as Table 2.

Variable	Men			Women		
	OR	(95% CI)	p-value	OR	(95% CI)	p-value
(Intercept)	**0.091**	**(0.068, 0.122)**	**<0.001**	**0.124**	**(0.096, 0.161)**	**<0.001**
**Individual**						
Age	**1.026**	**(1.023, 1.029)**	**<0.001**	**1.028**	**(1.026, 1.030)**	**<0.001**
Marital status (ref. not married nor in a civil union)	**0.753**	**(0.684, 0.830)**	**<0.001**	0.997	(0.935, 1.063)	0.920
Formal education (years)	**0.960**	**(0.944, 0.977)**	**<0.001**	0.989	(0.977, 1.002)	0.088
Religion (ref. no religion or other religion)						
Catholic	1.009	(0.909, 1.120)	0.869	1.174	**(1.045, 1.318)**	**0.007**
Protestant	0.969	(0.866, 1.084)	0.583	1.109	(0.986, 1.248)	0.085
Ethnicity (ref. not indigenous)	**1.244**	**(1.100, 1.408)**	**<0.001**	1.115	(0.999, 1.244)	0.052
Food insecurity (ref. has sufficient food)	**2.156**	**(1.996, 2.328)**	**<0.001**	**1.986**	**(1.873, 2.106)**	**<0.001**
**Household**						
Number of household members	0.995	(0.969, 1.022)	0.698	1.003	(0.982, 1.025)	0.770
% household members w/ depression	**1.425**	**(1.283, 1.582)**	**<0.001**	**1.298**	**(1.192, 1.414)**	**<0.001**
**Village**						
Village population	1.000	(0.999, 1.001)	0.490	1.000	(0.999, 1.000)	0.401
Village isolation (# access routes)	1.071	(0.996, 1.151)	0.064	1.064	(0.993, 1.140)	0.080
**Social network**						
Number of friends (degree)	0.995	(0.981, 1.008)	0.429	0.999	(0.988, 1.011)	0.911
% friends w/depression	**1.301**	**(1.097, 1.543)**	**0.002**	**1.350**	**(1.199, 1.520)**	**<0.001**
Number of adversaries (degree)	**1.082**	**(1.051, 1.113)**	**<0.001**	**1.093**	**(1.071, 1.115)**	**<0.001**
Average degree of friends	0.999	(0.981, 1.016)	0.889	0.998	(0.984, 1.011)	0.729
Triadic network characteristics[1]						
Negative triad (ref. balanced triad)	1.046	(0.928, 1.165)	0.118	1.071	(0.946, 1.196)	0.141
Incomplete triad (ref. balanced triad)	1.052	(0.918, 1.182)	0.528	**1.052**	**(1.006, 1.098)**	**0.003**
None (ref. balanced triad)	0.946	(0.779, 1.149)	0.574	0.877	(0.767, 1.003)	0.056
Village friend network density	1.050	(0.971, 1.135)	0.224	1.036	(0.970, 1.106)	0.290
Village adversary network density	1.057	(0.994, 1.123)	0.076	1.019	(0.967, 1.074)	0.483
**Wave of data collection**	1.019	(0.925, 1.122)	0.702	1.042	(0.966, 1.123)	0.287

Notes: ORs and 95% CIs are from mixed-effects logistic regression models fit to the same two-wave analytic sample used in the main manuscript, with adjustment for standard covariates (same as Table 2). Under this specification, each participant is assigned to one triad category under the hierarchy of negative > balanced > incomplete > no triad ('none'). These odds ratios compare each triadic categories to the reference of balanced triads. P-values are from Wald tests.

14. The results are interpreted clearly and reasonably, and limitations/caveats are acknowledged well. I do not have any major comments regarding the discussion.

Response: Thank you for your evaluation of our discussion and limitations sections.

15. "Individuals reporting food insecurity had an 85–222% increase in odds of depression or postpartum depression". I could not locate which statistics this was based upon. For postpartum depression the 95% CI were 2.04-5.09 for men, 1.44-2.37 for women. For all depression, it was 2.03-2.35 for men and 1.90-2.11 for women.

Response: We revised this section to report that the strongest association between depression and food insecurity was among postpartum men (OR=3.22, 95% CI 2.04-5.09), while the weakest association was among postpartum women (OR=1.85, 95% CI 1.44-2.37), now reflected on page 23.

<u>Reviewer: 4 Dr. Kenji Hashimoto, Chiba University Center for Forensic Mental Health</u>

These findings highlight the importance of examining structural properties of social networks beyond simple dyadic ties, as well as the need to include adversarial ties when studying the links between psychological health and face-to-face relationships in resource-limited settings. The findings of this study are interesting, and the manuscript is well written. I have two minor comments:

1. In this study, 34.97% of participants reported depressive symptoms, suggesting a higher prevalence compared with many other countries. Could socioeconomic factors (e.g., low income or lack of access to smartphones and digital communication) contribute to this higher prevalence?

Response: We appreciate your assessment of the study and the positive evaluation of our manuscript. We have incorporated your suggestions on adding socioeconomic context with regards to the high proportion of reported depression symptoms in our study population. The villages included in this study are small (range: 42-512 adults), traditional, mostly agricultural, and resource-limited, and 90% lack comprehensive health clinics. Mental health access is almost non-existent, and public utility services are intermittent, including electricity, water, and sanitation. This lack of health and basic public resources has been shown to be a

relevant determinant of mental health and exacerbates comorbid chronic conditions. Our participants have very scarce, if any, access to mental health providers (4). Recent systematic reviews in the Latin American context have associated these socioeconomic factors and systemic inequalities with a higher prevalence of depressive symptoms (5). We included these determinants of depression to the discussion (pg. 22-23), along with a reference to two recent studies that used an analogous measure in a similar rural Honduran population: One found that about 30% of the population had depressive symptoms, while the second one reported a 47% of depression prevalence—both proportions, as in our study, show significantly higher depression than other Latin American sites, including urban and rural areas (6). As we describe in our discussion and conclusion, studies focusing on these high-risk settings in Low- and Middle-Income Countries (LMIC) are historically underrepresented, largely due to logistical difficulties in accessing these populations and the cost of large-scale data collection in complex geographies and infrastructures. We hope that beyond our contributions to the literature of social networks and depression, we can also add to the available knowledge and social determinants of health of rural, resource-limited, and historically understudied communities that represent the global majority of the world's population.

2. The data were collected between October 2015 and July 2023. Did the COVID-19 pandemic influence the prevalence of depressive symptoms in this population?

Response: We thank you for your comment. Other reviewers also raised concerns about potential historical threats and methodological changes in Wave 4 of the parent RCT (fewer villages, no information on men in postpartum parents, different survey instruments). For clarity and methodological consistency in our results, per your and another reviewer's perspectives, we've decided to remove Wave 3 of our analysis (referred to as 'Wave 4' in the parent RCT) to avoid introducing methodological, sample change, or historical effects. In addition to the potential changes the pandemic might have introduced (which, in our evaluation, were minimal), the study population of the parent RCT was downsized from 176 villages to 82 in Wave 3 for exogenous reasons unrelated to this study. Furthermore, the instrument (survey design) was modified, and men were no longer asked about childbearing, among other modifications related to a new parent study. Though COVID case rates appeared low in the subset of the population measured within the parent RCT (only 4.5% of respondents reported having COVID between Waves 2 and 3, with only 46% of those cases diagnosed by a medical professional) and low within the study region per a national ecological analysis of COVID cases (1), we cannot rule out effects or confirm, the direction of the effects of the pandemic restrictions, as well as ecological effects of major hurricanes (Eta and Iota) that severely damaged the country at the end of 2020. Regarding depressive symptoms, we observed a decrease in the proportion of depressed individuals, which may have been related to economic rebound effects or the lifting of pandemic restrictions.

As the primary objective of this paper is to examine the relationship between social network structure and depression under relatively stable conditions, and to include postpartum men and women, we believe that objective is best achieved by limiting the sample to

homogeneous instruments and sample, and by using full information for all models, given that the additional wave was post-measurement, with a different purpose than the RCT parent study (2, 3). Evaluating whether the global 2020 COVID pandemic, through mechanisms such as lockdown-induced isolation or post-pandemic social reorganization, affected this relationship would require a distinct empirical design and research question beyond the scope of the present study.

We hope these clarifications align with all reviewers' suggestions, as we also include additional robustness checks for the key models in the main text. We appreciate this revision, which has improved the clarity and consistency of our methodology and highlighted the main findings and contributions of our study.

References

1. Escoto Rodríguez VC, Expósito Ruiz M. Variability of COVID-19 mortality in Honduras: influence of sociodemographic factors. International Journal for Equity in Health. 2025;24(1):60.
2. Beghini F, Pullman J, Alexander M, Shridhar SV, Prinster D, Singh A, et al. Gut microbiome strain-sharing within isolated village social networks. Nature. 2025;637(8044):167–75.
3. Feltham E, Forastiere L, Christakis NA. Cognitive representations of social networks in isolated villages. Nature human behaviour. 2025;9(8):1737–53.
4. González Y, Herrera IE, Moncada G. Prevalencia de trastornos mentales en relación con la prevalencia de enfermedades médicas crónicas en mayores de 18 años, departamento de Francisco Morazán y Olancho, año 2016. Portal de la Ciencia. 2018:158–74.
5. Errazuriz A, Avello-Vega D, Ramirez-Mahaluf JP, Torres R, Crossley NA, Undurraga EA, et al. Prevalence of depressive disorder in the adult population of Latin America: a systematic review and meta-analysis. The Lancet Regional Health–Americas. 2023;26.
6. Espinoza-Turcios E, Gonzales-Romero RM, Sosa-Mendoza C, Sierra-Santos M, Castro-Ramos HN, Zambrano LI, et al. Factors associated with hopelessness, depression and anxiety in the Honduran-Central America population during the COVID-19 pandemic. Frontiers in Psychiatry. 2023;14:1116881.
